# O_3_-Induced Priming Defense Associated With the Abscisic Acid Signaling Pathway Enhances Plant Resistance to *Bemisia tabaci*

**DOI:** 10.3389/fpls.2020.00093

**Published:** 2020-02-26

**Authors:** Honggang Guo, Yucheng Sun, Hongyu Yan, Chuanyou Li, Feng Ge

**Affiliations:** ^1^ State Key Laboratory of Integrated Management of Pest Insects and Rodents, Institute of Zoology, Chinese Academy of Sciences, Beijing, China; ^2^ CAS Center for Excellence in Biotic Interactions, University of Chinese Academy of Sciences, Beijing, China; ^3^ College of Bioscience and Resource Environment/Key Laboratory of Urban Agriculture (North China), Ministry of Agriculture and Rural Affairs of the People's Republic of China, Beijing University of Agriculture, Beijing, China; ^4^ State Key Laboratory of Plant Genomics, National Center for Plant Gene Research, Institute of Genetics and Developmental Biology, Chinese Academy of Sciences, Beijing, China

**Keywords:** elevated O_3_, abscisic acid, callose, priming defense, *Bemisia tabaci*

## Abstract

Elevated ozone (O_3_) modulates phytohormone signals, which subsequently alters the interaction between plants and herbivorous insects. It has been reported that elevated O_3_ activates the plant abscisic acid (ABA) signaling pathway, but its cascading effect on the performance of herbivorous insects remains unclear. Here, we used the ABA-deficient tomato mutant *notabilis* (*not*) and its wild type, Ailsa Craig (AC), to determine the role of ABA signaling in mediating the effects of elevated O_3_ on *Bemisia tabaci* in field open-top chambers (OTCs). Our results showed that the population abundance and the total phloem-feeding duration of *B. tabaci* were decreased by O_3_ exposure in AC plants compared with *not* plants. Moreover, elevated O_3_ and *B. tabaci* infestation activated the ABA signaling pathway and enhanced callose deposition in AC plants but had little effect on those in *not* plants. The exogenous application of a callose synthesis inhibitor (2-DDG) neutralized O_3_-induced resistance to *B. tabaci*, and the application of ABA enhanced callose deposition and exacerbated the negative effects of elevated O_3_ on *B. tabaci*. However, the application of 2-DDG counteracted the negative effects of O_3_ exposure on *B. tabaci* in ABA-treated AC plants. Collectively, this study revealed that callose deposition, which relied on the ABA signaling pathway, was an effective O_3_-induced priming defense of tomato plants against *B. tabaci* infestation.

## Introduction

The global tropospheric ozone (O_3_) concentration has increased from less than 10 ppb in the preindustrial era to 35-50 ppb in the present day in the Northern Hemisphere (Ainsworth et al., 2012) and is predicted to increase at a rate of approximately 0.5-2% per year in some regions, such as East Asia (Ohara et al., 2007; IPCC, 2013; Cooper et al., 2014). Tropospheric O_3_ is an important atmospheric pollutant and a greenhouse gas that causes changes in plant metabolism, including the hormone signaling pathway (Ashmore, 2005; Gupta et al., 2005). The alterations in plant biochemistry under elevated O_3_ affect the quality and palatability of plant tissue and therefore changes in resistance to infestation by herbivorous insects (Peltonen et al., 2010).

Upon pre-exposure to abiotic stress, plants are able to establish a defense priming with a fast and/or strong activation of defense responses, including phytohormones, against subsequent challenges from microbes, insects, or other biotic stresses (Mauch-Mani et al., 2017; Hilker and Schmülling, 2019). Previous studies have shown that because of strong oxidative stress, pre-exposure to O_3_ can prime tomato leaves for an enhanced defense against subsequent herbivorous insect infestation by upregulating the expression of salicylic acid (SA)-dependent defensive gene *pathogenesis-related protein* and increasing the emission of jasmonic acid (JA)-mediated monoterpene volatiles by tomato plants to decrease the population abundance and feeding fitness of *B. tabaci* (Cui et al., 2012; Cui et al., 2014). In addition to the SA and JA signaling pathways, O_3_ pre-exposure activates the abscisic acid (ABA) signaling pathway, with a significant increase in ABA accumulation *via* the direct oxidation of the ABA precursor xanthoxin and the expression of ABA-related genes in some species, such as Arabidopsis, tomato, and Chinese pine (Li et al., 2011; McAdam et al., 2017). ABA is an important signal in regulating phloem-sucking insect infestation (Kerchev et al., 2013; Hillwig et al., 2016). For example, a Y-tube olfactometer experiment has discovered that *B. tabaci* prefers the ABA-deficient mutant *sitiens* to wild-type Rheinlands Ruhm (RR) plants, implying a potential role for ABA signaling in plant resistance to *B. tabaci* (Pérez-Hedo et al., 2015). Furthermore, a recent study has revealed that the drought-induced ABA signaling pathway enhances the mesophyll/phloem resistance of *Medicago truncatula*, reducing the feeding efficiency of pea aphids (*Acyrthosiphon pisum*) (Guo et al., 2015), which suggests that ABA signaling is required for priming the defense of plants against phloem-sucking insects.

ABA signaling positively regulates plant resistance to biotic stresses by accelerating the accumulation of callose in host plants (Flors et al., 2005; Luna et al., 2011). Treatment with exogenous ABA increases callose deposition in Arabidopsis (*Arabidopsis thaliana*), which improves the resistance of the plant to the plant pathogen *Plectosphaerella cucumerina* (García-Andrade et al., 2011; Mauch-Mani et al., 2017). Furthermore, ABA positively regulates starch amylase (*BAM1*) and suppresses beta-1,3-glucanase (*PR2*), leading to augmented callose deposition to defend against pathogen infection (Oide et al., 2013; Gamir et al., 2018). Likewise, callose accumulation is effective for plant resistance to phloem-sucking insects (Cheng et al., 2013; Liu et al., 2017; Yao et al., 2019). When attacked by brown planthoppers (*Nilaparvata lugens* Stål), callose deposition is activated around sieve plates in rice (*Oryza sativa*), which is a disadvantage for the fitness of brown planthoppers (Hao et al., 2008). Given that callose accumulation is implicated in ABA-regulated resistance to insects, O_3_-induced upregulation of the ABA signaling pathway presumably increases callose deposition, which subsequently provides relatively strong phloem resistance to phloem-sucking insects.

*B. tabaci* is a phloem-sucking insect that is regarded as the most destructive and agriculturally invasive pest in China. *B. tabaci* causes extensive crop losses annually, estimated at billions of dollars, directly through feeding and through virus transmission (Dalton, 2006; De Barro et al., 2011). Understanding the physiological basis of the effects of climate change on invasive insects is crucial to crop production health and security. Here, we hypothesized that elevated O_3_ increased ABA signal-regulated callose deposition, which could be detrimental to the population abundance and feeding efficiency of *B. tabaci*. To experimentally test this hypothesis, we used *notabilis* (*not*, an ABA-deficient mutant of *Lycopersicon esculentum*) and its wild type, Ailsa Craig (AC, background of *not*), to determine the effects of elevated O_3_ on the ABA signaling pathway of the tomato plant and its cascading effect on the performance of *B. tabaci*. Our specific objectives were to determine (1) whether elevated O_3_ activates the ABA signaling pathway; (2) whether O_3_-induced upregulation of the ABA signaling pathway causes further accumulation of callose; and (3) whether ABA signaling is involved in regulating the effects of elevated O_3_ on the feeding behavior and population abundance of *B. tabaci*.

## Materials and Methods

### Treatments Under Different O_3_ Concentrations

Experiments were performed in eight octagonal, open-topped chambers (OTCs) (2.1 m diameter and 2 m height) in the field at the Observation Station of the Global Change Biology Group, Institute of Zoology, Chinese Academy of Science in Xiaotangshan County, Beijing, China (40°11′N, 116°24′E). The conditions under which the O_3_ concentrations were set were as follows: (i) current atmospheric O_3_ levels (42 ± 3.8 ppb); (ii) elevated O_3_ levels (89 ± 5.3 ppb). Four blocks were used for the O_3_ treatment, and each block contained paired OTCs, one with ambient O_3_ and one with elevated O_3_.

For elevated O_3_ treatment, O_3_ was generated from ambient air by an O_3_ generator (3S-A15, Tonglin Technology, Beijing, China) and then transported to the entrances of the OTCs using a fan (HB-429, 4.1 m^3^ min^-1^, Ruiyong Mechanical and Electrical Equipment Company, Beijing, China). Mixed air (O_3_ and ambient air) was ventilated into each OTC through columniform polyvinyl chloride pipes (inner diameter 11 cm, outer diameter 16 cm). OTCs were ventilated with air daily from 9:00 a.m. to 5:00 p.m. through a hemispherical stainless-steel sprayer (diameter = 30 cm) situated 0.5 m above the canopy at a rate of approximately 15 m^3^ min^-1^, resulting in approximately two air changes min^-1^ in each OTC. O_3_ concentrations were monitored (AQL-200, Aeroqual, New Zealand) within the OTCs four times per day throughout the studies to maintain relatively stable O_3_ concentrations. The measured O_3_ concentrations throughout the experiment (mean ± SD d^-1^) were 42 ± 3.8 ppb in the ambient O_3_ chambers and 89 ± 5.3 ppb in the elevated O_3_ chambers. Air temperatures were measured and did not differ significantly between the two treatments (22.7 ± 1.9°C in OTCs with ambient O_3_ vs. 24.2 ± 2.0°C in OTCs with elevated O_3_).

### Host Plants and Insects

The *not* (LA3614; cv. AC background) tomato plants, ABA-deficient mutants with a dominant mutation in the *NCED1* gene, which encodes *9-cis-epoxycarotenoid dioxygenase* (NCED), a rate-limiting enzyme of ABA biosynthesis (Thompson et al., 2004), were kindly provided by Professor Chuanyou Li (Institute of Genetics and Developmental Biology at the Chinese Academy of Sciences). The *not* and wild-type AC seeds were placed in petri dishes containing 0.75% agar and kept under natural lighting at 25°C for 2 days until germination. The germinated seeds were individually sown into approximately 1.5 L small pots (one plant per pot) and maintained in an unstressed state by keeping the relative humidity at 80 to 90%. The host plants were cultivated until the 2-3-leaf stage for the subsequent experiments.

Tomato plants were maintained in the OTCs for 43 days from seedlings with two to three leaves to the end of the experiment. Insecticides were not used throughout the experiment. The plants were irrigated every 2 days. Pot placement was rerandomized within each OTC once every week. After the plants had been in the OTCs for 18 days, they were used for further experiments. For all biochemical, molecular, and histochemical analyses, leaflets from fully expanded middle-aged leaves were used.

The *B. tabaci* Middle East Asia Mino 1 genetic group, also called the B biotype, was kindly provided by Professor Youjun Zhang (Department of Plant Protection, Institute of Vegetables and Flowers, Chinese Academy of Agricultural Sciences, Beijing 100081, China). *B. tabaci* was initially transferred to cotton plants to maintain the population in separate cages in a greenhouse at 25 ± 2°C and 75 ± 10% relative humidity, with a 14-h light/10-h dark photoperiod. The purity of the colony was controlled by sampling 30 adults and sequencing the mitochondrial cytochrome oxidase subunit I *(mtCOI*, GenBank Accession No. GQ332577) gene, which is a molecular marker that distinguishes the different *B. tabaci* groups (De Barro et al., 2011). The cotton plants were grown in the greenhouse under the same conditions in which the herbivores were reared.

### Plant Growth Analysis and Leaf Gas Exchange

After 18 days of O_3_ fumigation, 240 tomato plants in total in eight OTCs, which contained 30 tomato plants (15 AC plants and 15 *not* plants) with uniform size per OTC, were randomly selected for determination of the biomass and the number of stippled leaves, burned leaves, curled leaves, and deciduous leaves. Leaves from the third or fourth branch (counting from the top) of each plants (8 AC plants and 8 *not* plants) per chamber were randomly selected to determine the net photosynthetic rate and stomatal conductance (g_s_) under light conditions with a Li-Cor 6400 gas exchange system (6400-40; Li-Cor Inc., Lincoln, NE, USA) (**Figure S.1A**). The CO_2_ concentration was maintained at 400 µmol mol^−1^. Before gas exchange was measured, illumination was set to 90% red and 10% blue, and the temperature was set to 25°C. The photosynthetic photon flux density (PPFD) was fixed at a saturating intensity of 1200 µmol m^−2^ s^−1^. Measurements were taken when the CO_2_ assimilation rate was stable for at least 2 min.

### Reactive Oxygen Species (ROS) Accumulation

After 18 days of O_3_ fumigation, leaves from the third or fourth branch (counting from the top) of each plant (8 AC plants and 8 *not* plants) per OTC were randomly selected to measure ROS content according to a modified method described previously (Guo et al., 2018) (**Figure S.1A**).

### Scanning Electron Microscopy of Stomatal Apertures

After 18 days of O_3_ fumigation, leaves from the third or fourth branch (counting from the top) of each plant (5 AC plants and 5 *not* plants) per OTC were fixed in 2.5% glutaraldehyde in 1 mM sodium phosphate buffer (pH 6.8) for 12 h and then washed in 50, 60, 70, 90, and 100% ethanol (15 min for each wash) (**Figure S.1A**). The samples were subjected to critical-point drying in CO_2_ and were coated with gold using an Eiko 1B.5 sputter coater (Eiko, Tokyo, Japan). The samples were then examined with a Hitachi s570 scanning electron microscope (SEM, Tokyo, Japan) at an accelerating voltage of 5-15 kV. Three-four leaves per plant were examined with SEM. Five SEM fields of view scope (accumulated 60 stomata) were randomly selected to take images for analysis of stomatal apertures using Image-Pro Plus. The stomatal apertures were recorded by the ratio of length to width.

### *Bemisia tabaci* Infestation

Plants were arranged for two different treatments with *B. tabaci*. After 18 days of O_3_ fumigation, 192 tomato plants in total in eight OTCs, which contained 24 tomato plants (12 AC plants and 12 *not* plants) of uniform size per OTC, were randomly selected for the 21-day *B. tabaci* infestation experiment (**Figure S.1B**). Leaves from the third or fourth branch (counting from the top) of each plant were inoculated with five pairs of newly emerging *B. tabaci*, which were maintained in a clip-cage to develop and produce offspring on tomato plants for 3 weeks. All *B. tabaci* stages (eggs, one to four nymphs and adults) per plant were included in the quantification of *B. tabaci* abundance.

In the second part of this experiment, 128 tomato plants in total in eight OTCs, 16 tomato plants (eight AC plants and eight *not* plants) of uniform size per OTC, were randomly selected after 39 days of O_3_ fumigation. Leaves from the third or fourth branch (counting from the top) of each plant were damaged with ten pairs of newly emerging *B. tabaci*. *B. tabaci*, which were maintained in a clip-cage, infested freely for 24 h. Another 128 tomato plants in total in eight OTCs, which included 16 tomato plants (eight AC plants and eight *not* plants) of uniform size per OTC, were randomly selected as control plants, and their corresponding leaves were caged in the same way but without *B. tabaci* (**Figure S.1C**). Leaves from each plant were harvested separately after 24 h of *B. tabaci* infestation and were immediately stored in liquid N for the determination of ABA content, callose content, the enzymatic activity of callose synthase and beta-1,3 glucanase, and the relative expression of ABA signaling-related genes callose synthase-related genes and callose degradation-related genes (details are reported below).

### Feeding Behavior of *B. tabaci*

A total of 80 tomato plants in eight OTCs, 10 tomato plants (five AC plants and five *not* plants) of uniform size per OTC, were randomly selected as host plants to evaluate *B. tabaci* feeding behaviors after 39 days of O_3_ fumigation. Fifteen effective replicates were performed for statistical analyses. *B. tabaci* feeding behavior was monitored using the electrical penetration graph (EPG) method (Liu et al., 2013; Tan et al., 2017). The EPG system was placed into an electrically grounded Faraday cage to prevent external electrical noise. The EPG signals were digitized with a DI710-UL analog-to-digital converter, and the output was acquired and stored with PROBE 3.4 software. The data were subsequently analyzed with STYLET 2.0 software. Phases of feeding behavior were described by EPG parameters related to non-penetration (NP), pooled pathway phase activities (C), salivary secretion into sieve elements (E1), phloem ingestion (E2), derailed stylets (F), and xylem ingestion (G) were extracted from each recording and compared among treatments. Twelve hours of EPGs were continuously recorded for each replicate. All experiments were carried out under artificial light (1,500 lx) with a 16-h light/8-h dark regime at 25°C ± 2°C and 70% relative humidity (RH).

### Exogenous ABA and 2-Deoxy-D-Glucose Treatment of Tomato Plants

During O_3_ exposure, 800 AC plants in total in eight OTCs, which contained 100 AC plants of uniform size per OTC, were randomly selected. Fifty AC plants were treated with a final concentration of 100 µM ABA in 0.5% ethanol (Du et al., 2014; Pérez-Hedo et al., 2015), and 50 AC plants were treated with H_2_O, which was considered the control treatment. Both sides of leaves were sprayed with the prepared chemical reagents once every three days at 8:00 a.m.

Before 24-h *B. tabaci* infestation, 25 AC plants with ABA treatment and 25 AC plants with H_2_O treatment of each OTC (400 AC plants in total in eight OTCs) were randomly selected to be injected with a final concentration of 25 mM 2-deoxy-D-glucose (2-DDG) (Asselbergh and Höfte, 2007) (**Figures S.1D, E**). According to the method described in Xiao et al. (2018), three tomato leaves from the third or fourth branch (counting from the top) of each plant were randomly selected to be injected with callose inhibitor (2-deoxy-D-glucose), each leaf having three injection sites. The callose inhibitor (2-deoxy-D-glucose) was injected through two rubber stoppers, which were squeezed together. The solution penetrated the leaf through the stomata and rapidly infiltrated both ends of the injection site. Generally, two to three injections were enough to fill the gap between the veins on both sides of the leaf.

### Callose Content and Enzyme Activity

After 39 days of O_3_ fumigation, leaves from the third or fourth branch (counting from the top) of tomato plants with and without *B. tabaci* infestation were collected for callose content and enzyme activity assays. Callose content was measured as described previously, with minor modifications (Zhang et al., 2015). In brief, the leaves were fixed and dehydrated in ethanol and homogenized in 1 M NaOH for 2 min and then transferred to 1.5 ml Eppendorf tubes. To dissolve the callose, the homogenate was incubated in a water bath (80°C, 30 min) and then centrifuged (12,000 rpm, 15 min) at room temperature. A volume of 200 μl of supernatant including callose was mixed with a 1.25-ml aniline blue mixture (3:1 0.1% aniline blue:1 M glycine, pH 9.5) in a water bath (50°C, 20 min). Callose was quantified by fluorescence spectrophotometry using a SpectraMax *i3* (Bio-Rad, Hercules, CA) at an excitation wavelength of 400 nm and an emission wavelength of 500 nm, using laminarin as a standard callose source. Callose synthase activity and beta-1,3-glucanase activity were assayed according to the method described by Zhang et al. (2015), with some modifications.

### ABA Quantification

After 39 days of O_3_ fumigation, leaves from the third or fourth branch (counting from the top) of tomato plants with and without *B. tabaci* infestation were collected for ABA measurements. Approximately 300 mg of fresh leaves were used to analyze the ABA content according to a modified method that was described previously (Guo et al., 2018). Plant tissue was homogenized in liquid nitrogen and sealed in 10 ml tubes. Extraction buffer (0.5 ml) was added to each sample. The samples were agitated for 30 min at 4°C. Subsequently, 1 ml of CH_2_Cl_2_ was added, and the samples were agitated for another 30 min at 4°C. The samples were then centrifuged at 13,000 g for 10 min. After centrifugation, two phases formed, with the plant debris located in the middle of the two layers. The aqueous phase was discarded, and approximately 1.5 ml of the lower layer was collected. Then, the samples were concentrated in a dry machine and resolubilized in 200 µl of MeOH. Before transfer to a glass tube, the sample was filtered through a 0.22 µm filter.

A Perkin-Elmer 200 liquid chromatograph coupled with an Analytical Biosystems Sciex API 4000 mass spectrometer, with a triple quadrupole and turbo spray ion source, was used. Mass spectrometric experimental conditions were as follows: Q2 gas pressure, 3.7 × 10^-5^ Torr; Q1 and Q3 resolution, 0.7 amu; cycle time, 605 ms (11 transitions with a dwell time of 50 ms); spray voltage, 5.5 kV; sheath gas flow rate, 55 ml min^-1^; auxiliary gas flow rate, 20 ml min^-1^; auxiliary gas temperature, 400°C. Air was used as sheath and auxiliary gas. A Symmetry Waters C18 column (2.1 × 50 mm, 5 µm particle diameter) was used, and gradient chromatographic separation was performed at a flow rate of 0.2 ml min^-1^ as follows: 5% (1 min) to 95% (5 min) to 95% (7 min) to 5% (7.1 min) to 5% (15 min) of eluent A/eluent B. Eluent A was 0.1% HCOOH. Eluent B was 100% acetonitrile. A 5-µl volume of the sample was injected into a column for analysis. The concentrations of the hormones were estimated using standard curves, which were constructed based on a gradient dilution of the reference phytohormone (Agilent Chemical Co.).

### Gene Expression

RNA extraction and quantitative PCR gene expression were measured using quantitative reverse transcription polymerase chain reaction. Each treatment was replicated with four biological repeats and four technical repeats. The RNeasy Mini Kit (Qiagen, Dusseldorf, Germany) was used to isolate total RNA from the leaves (0.05 g from samples stored at -70°C), and 1 μg of RNA was used to generate cDNA. We used real-time quantitative PCR (qPCR) to determine the mRNA levels according to a modified method that was described previously (Guo et al., 2018). Specific primers for each gene were designed from the expressed sequence tag sequences using Primer 5 software (**Table S.7**). The qPCRs were performed using the following protocol: a 20 μl total reaction volume including 10 μl of 2× SYBR Premix EX Taq^™^ (Qiagen, Dusseldorf, Germany) Master Mix, 5 mM of each gene-specific primer, and 1 μl of cDNA template. Reactions were carried out using the Mx 3000P detection system (Stratagene) as follows: 2 min at 94°C; followed by 40 cycles of 20 s at 95°C, 30 s at 56°C, and 20 s at 68°C; and finally one cycle of 30 s at 95°C, 30 s at 56°C, and 30 s at 95°C (Guo et al., 2015). We used *TIP41* and actin as internal qPCR standards; every target gene's expression level was normalized to the tomato *TIP41* and actin gene (Expósito-Rodríguez et al., 2008).

### Statistical Analysis

All statistical analyses were performed with the statistical package IBM SPSS Statistics 21.0. A split-split plot design was used for quantifying the population abundance and feeding behavior of *B. tabaci*, callose content, activity of callose synthase and beta-1,3-glucanase, ABA content, and relative expression of *NCED1*, *Sucrose non-fermenting 1-related protein kinase 2* (*SnRK2*), *callose synthase gene 11* (*Cals11*), *callose synthase gene 12* (*Cals12*), and *beta-1,3-glucanase* gene, for which O_3_ and block (a pair of ambient and elevated OTCs) were the main effects, *B. tabaci* infestation constituted the subplot effect, and tomato genotype constitute the sub-subplot effect. The main effects of O_3_, *B. tabaci* infestation, and tomato genotype on plant were tested according to the following model:

$${\text{X}}_{ijklm}=\mu +O_{i}+B\;{\left(O\right)}_{j(i)}+G_{k}+OG_{ik}+GB{\left(O\right)}_{kj(i)}+W_{l}+OW_{il}+WB{\left(O\right)}_{lj(i)}+GWB{\left(O\right)}_{klj(i)}+{\text{e}}_{m(ijkl)}$$

where O is the O_3_ treatment (*i* = 2), B is the block (*j* = 4), G is the tomato genotype (*k* = 2), and *W* is the *B. tabaci* infestation (*l* = 2). X*_ijklm_* represents the error because of the smaller scale differences between samples and variability within blocks (SPSS 21.0, SPSS Inc., Chicago, IL, USA). Effects were considered significant if *P* < 0.05. Tukey's multiple range tests were used to separate means when ANOVAs were significant (*P* < 0.05).

## Results

### Elevated O_3_ Negatively Affected the Performance of *B. tabaci*

**Figure 1:**
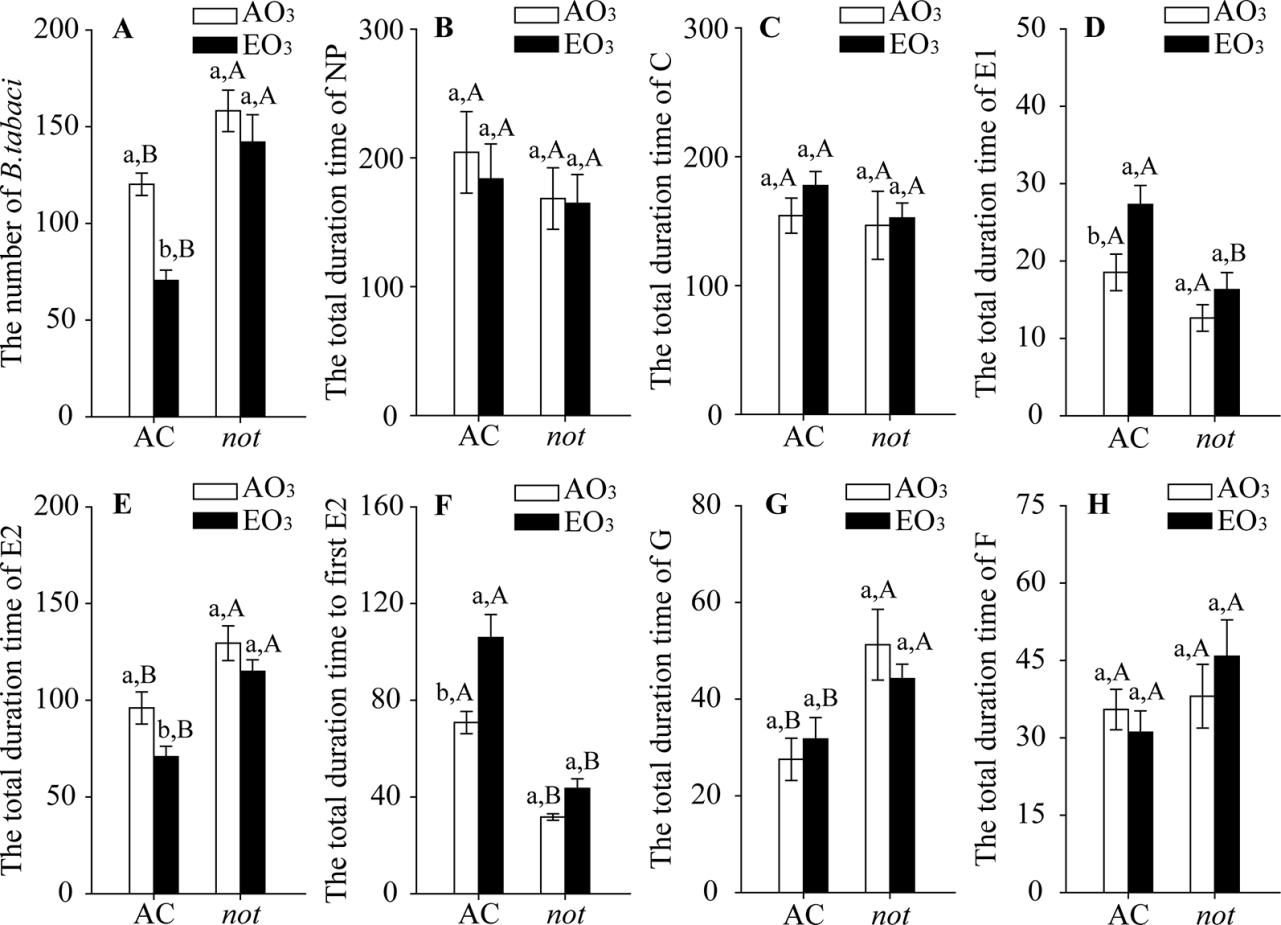
Population abundance and feeding behavior of *Bemisia tabaci* when fed on wild-type (AC) and ABA-deficient mutants (*not*), grown under ambient O_3_ (AO_3_) and elevated O_3_ (EO_3_). **(A)** Population abundance (number per plant). Each value represents the average (± SE) of four OTCs (12 plants for each genotype per OTC). **(B)** ‘Nonpenetration' (NP, stylets are outside the plants), **(C)** ‘pathway' (C, mostly intramural probing activities between mesophyll or parenchyma cells), **(D)** ‘salivation' (E1, salivary secretion into sieve elements), **(E)** ‘phloem ingestion' (E2, ingesting the phloem sap), **(F)** total time to first E2, **(G)** ‘xylem ingestion' (G, stylet penetration of tracheary elements), and **(H)** derailed stylets (F, stylets are exhibiting penetration difficulties). Values are the mean (± SE) of 15 biological replicates. Different lowercase letters indicate significant differences between ambient O_3_ and elevated O_3_ within the same genotype. Different uppercase letters indicate significant differences between genotypes within the same O_3_ treatment, as determined by Tukey's multiple range test at *P* < 0.05.

Elevated O_3_ decreased the population abundance of *B. tabaci* by 41% on the AC plants but did not affect those on the *not* plants. Regardless of O_3_ concentration, *B. tabaci* had more abundant populations on the *not* plants than on the AC plants (**Figure 1A**). For the feeding behavior of *B. tabaci*, elevated O_3_ increased the total duration of salivating into sieve elements (E1 phase) by 47%, decreased the total duration of phloem ingestion (as indicated by E2 phase) by 26%, and prolonged the total time to the first E2 by 50% when reared on the AC plants, but the total duration of E1, the total duration of E2, and the total time to the first E2 were not affected by elevated O_3_ when *B. tabaci* was reared on the *not* plants. The *B. tabaci* had a shorter E1 phase and total time to the first E2, but a longer E2 phase and G phase on *not* plants than on AC plants under both O_3_ concentrations (**Figures 1B–H**; **Table S.1**).

### Elevated O_3_ Negatively Affected Plant Growth

**Figure 2:**
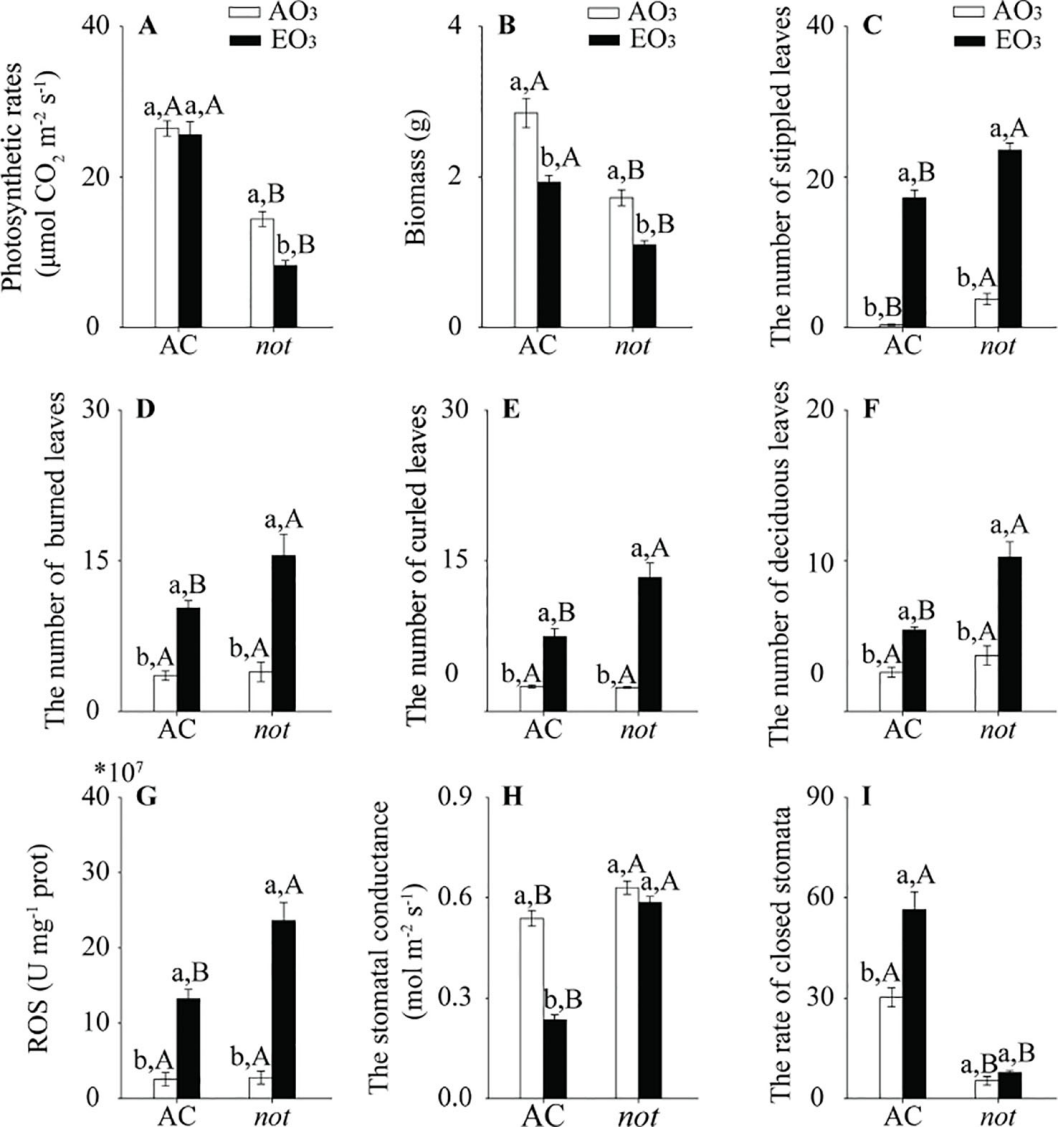
Growth traits of two tomato genotypes (AC and *not*) grown under ambient O_3_ (AO_3_) and elevated O_3_ (EO_3_) without *B. tabaci* infestation. **(A)** Photosynthetic rate, **(B)** biomass, **(C)** stippled leaves, **(D)** burned leaves, **(E)** curled leaves, **(F)** deciduous leaves, **(G)** ROS, **(H)** stomatal conductance (g_s_), and **(I)** rate of closed stomata. Each value represents the average (± SE) of four OTCs (15 plants for each genotype per OTC). Different lowercase letters indicate significant differences between ambient O_3_ and elevated O_3_ within the same genotype. Different uppercase letters indicate significant differences between genotypes with the same O_3_ treatment, as determined by Tukey's multiple range test at *P* < 0.05.

Regardless of plant genotype, elevated O_3_ had little effect on photosynthetic rate and reduced the biomass of plants but increased stippled leaves, burned leaves, curled leaves, deciduous leaves, and ROS accumulation in AC plants. Furthermore, the negative effects of elevated O_3_ on those growth traits were more severe in the *not* plants than on the AC plants (**Figures 2A–G**; **Table S.2**). With respect to stomatal parameters, the stomatal conductance was decreased by 44% when AC plants were grown under elevated O_3_, and the rate of closed stomata was increased by 86%. By contrast, elevated O_3_ did not affect the stomatal conductance or the rate of closed stomata in *not* plants (**Figures 2H, I**; **Table S.2**).

### Elevated O_3_ Activated the SA Signaling Pathway, But Had Little Effect on the JA Signaling Pathway

**Figure 3:**
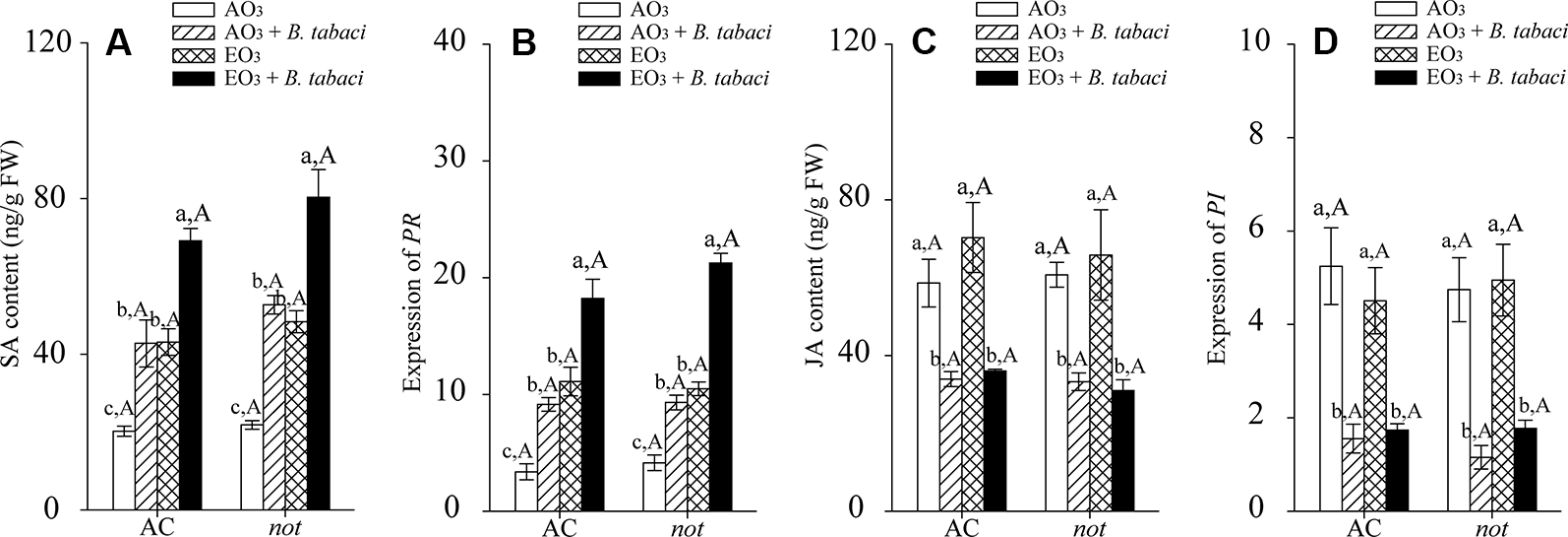
SA content, JA content, and fold-change in the expression of related genes involved in the SA- and JA-dependent signaling pathways for two tomato genotypes grown under ambient O_3_ (AO_3_) and elevated O_3_ (EO_3_) with and without *B. tabaci* infestation. **(A)** SA content; **(B)** relative expression of *PR*; **(C)** JA content; **(D)** relative expression of *PI*. Each value represents the mean (± SE) of four OTCs (eight plants for each genotype per OTC). Different lowercase letters indicate significant differences among the combinations of *B. tabaci* treatment and O_3_ concentrations within the same genotype. Different uppercase letters indicate significant differences between genotypes within the same O_3_ treatment and *B. tabaci* treatment, as determined by Tukey's multiple range test at *P* < 0.05.

*B. tabaci* infestation and elevated O_3_, individually and combined, significantly activated the SA signaling pathway in terms of SA contents and the relative expression of *pathogenesis-related protein* (*PR*) in the AC and *not* plants. Regardless of O_3_ concentration and *B. tabaci* infestation, the SA content and the relative expression of foliar *PR* were equivalent in AC and *not* plants. Elevated O_3_ had little effect on the foliar JA accumulation and the relative expression level of *proteinase inhibitor* (*PI*) in AC and *not* plants with and without *B. tabaci* infestation. Under both O_3_ concentrations, the foliar JA concentration and the relative expression level of *PI* were reduced by *B. tabaci* infestation and were not affected by plant genotype (**Figure 3**; **Table S.3**).

### Exposure to Elevated O_3_ Activated the ABA Signaling Pathway

**Figure 4:**
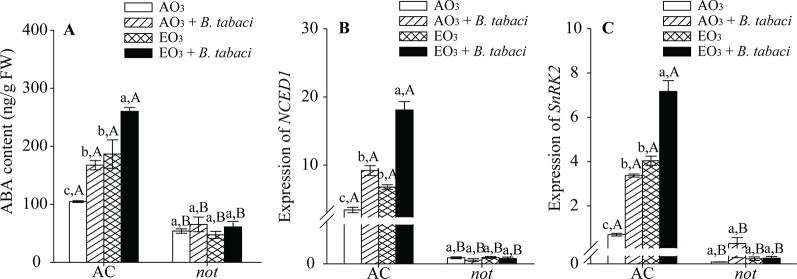
ABA content and relative expression of related genes involved in the ABA signaling pathway for two tomato genotypes grown under ambient O_3_ (AO_3_) and elevated O_3_ (EO_3_) with and without *B. tabaci* infestation. Each value represents the average (± SE) of four OTCs (eight plants for each genotype per OTC). **(A)** ABA content, **(B)** relative expression of *NCED1*, and **(C)** relative expression of *SnRK2*. Different lowercase letters indicate significant differences among the combinations of *B. tabaci* treatment and O_3_ concentrations within the same genotype. Different uppercase letters indicate significant differences between genotypes within the same O_3_ treatment and *B. tabaci* treatment, as determined by Tukey's multiple range test at *P* < 0.05.

*B. tabaci* infestation and elevated O_3,_ individually and combined, significantly increased the ABA signaling pathway in terms of ABA contents and the relative expression of *NCED1* and *SnRK2* in the AC plants but had little effect on the ABA signaling pathway in the *not* plants. Regardless of O_3_ concentration and *B. tabaci* infestation, the ABA content and the relative expression of *NCED1* and *SnRK2* were significantly higher in the AC plants than in the *not* plants (**Figure 4**; **Table S.3**).

### Elevated O_3_ Induced Callose Deposition

**Figure 5:**
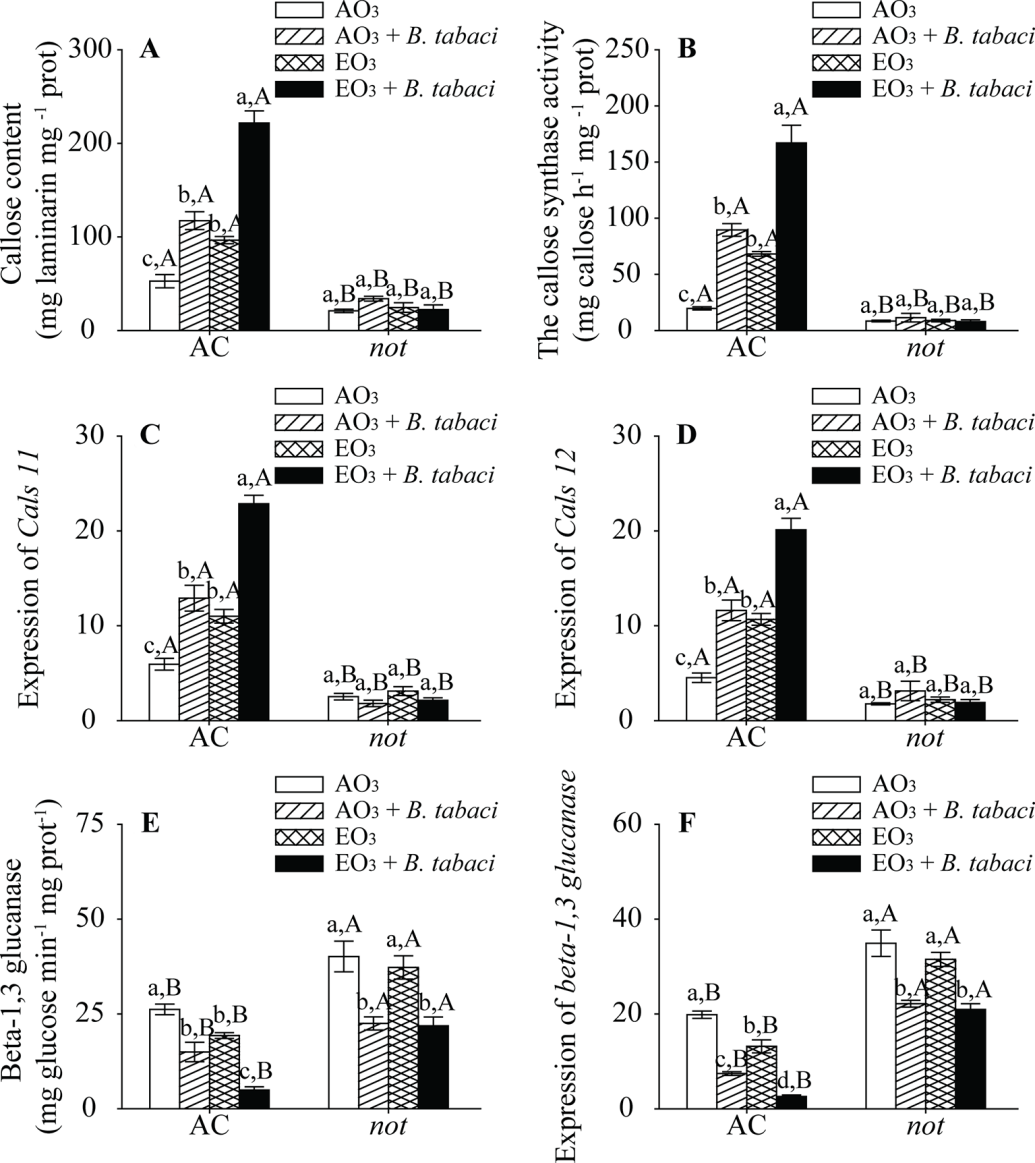
Callose content, enzyme activity, and fold-change in the expression of related genes involved in the callose synthesis and degradation for two tomato genotypes grown under ambient O_3_ (AO_3_) and elevated O_3_ (EO_3_) with and without *B. tabaci* infestation. **(A)** Callose content, **(B)** callose synthase activity, **(C)** relative expression of *Cals 11* gene, **(D)** relative expression of *Cals 12* gene, **(E)** beta-1,3 glucanase activity, and **(F)** relative expression of beta-1,3 glucanase gene. Each value represents the average (± SE) of four OTCs (eight plants for each genotype per OTC). Different lowercase letters indicate significant differences among the combinations of *B. tabaci* treatment and O_3_ concentrations within the same genotype. Different uppercase letters indicate significant differences between genotypes within the same O_3_ treatment and *B. tabaci* treatment, as determined by Tukey's multiple range test at *P* < 0.05.

O_3_ exposure, *B. tabaci* infestation, and plant genotype significantly affected the content of foliar callose. *B. tabaci* infestation increased the content of foliar callose in the AC plants under both O_3_ concentrations. Regardless of *B. tabaci* infestation, the callose content was nearly 2-fold higher in the AC plants under elevated O_3_ than under ambient O_3_. By contrast, elevated O_3_ and *B. tabaci* infestation, individually and combined, did not affect the callose content of *not* plants. The AC plants had higher callose content than the *not* plants (**Figure 5A**). Furthermore, the enzyme activity of callose synthase and the relative expression of *Cals11* and *Cals12*, which are related to callose synthase, were consistent with the content of foliar callose, as they were upregulated by elevated O_3_ and *B. tabaci* infestation individually and combined in the AC plants but unaffected in the *not* plants (**Figures 5B–D**).

We also found that the activities of the callose degradation enzyme beta-1,3 glucanase and its transcripts were decreased by elevated O_3_ and *B. tabaci* infestation in the AC plants. However, elevated O_3_ had no effects on these in the *not* plants. The AC plants had lower enzyme activity and transcripts of beta-1,3 glucanase than *not* plants (**Figures 5E, F**; **Table S.4**).

### The ABA Signaling Pathway Positively Regulated O_3_-Induced Callose Deposition to Combat *B. Tabaci* Infestation

**Figure 6:**
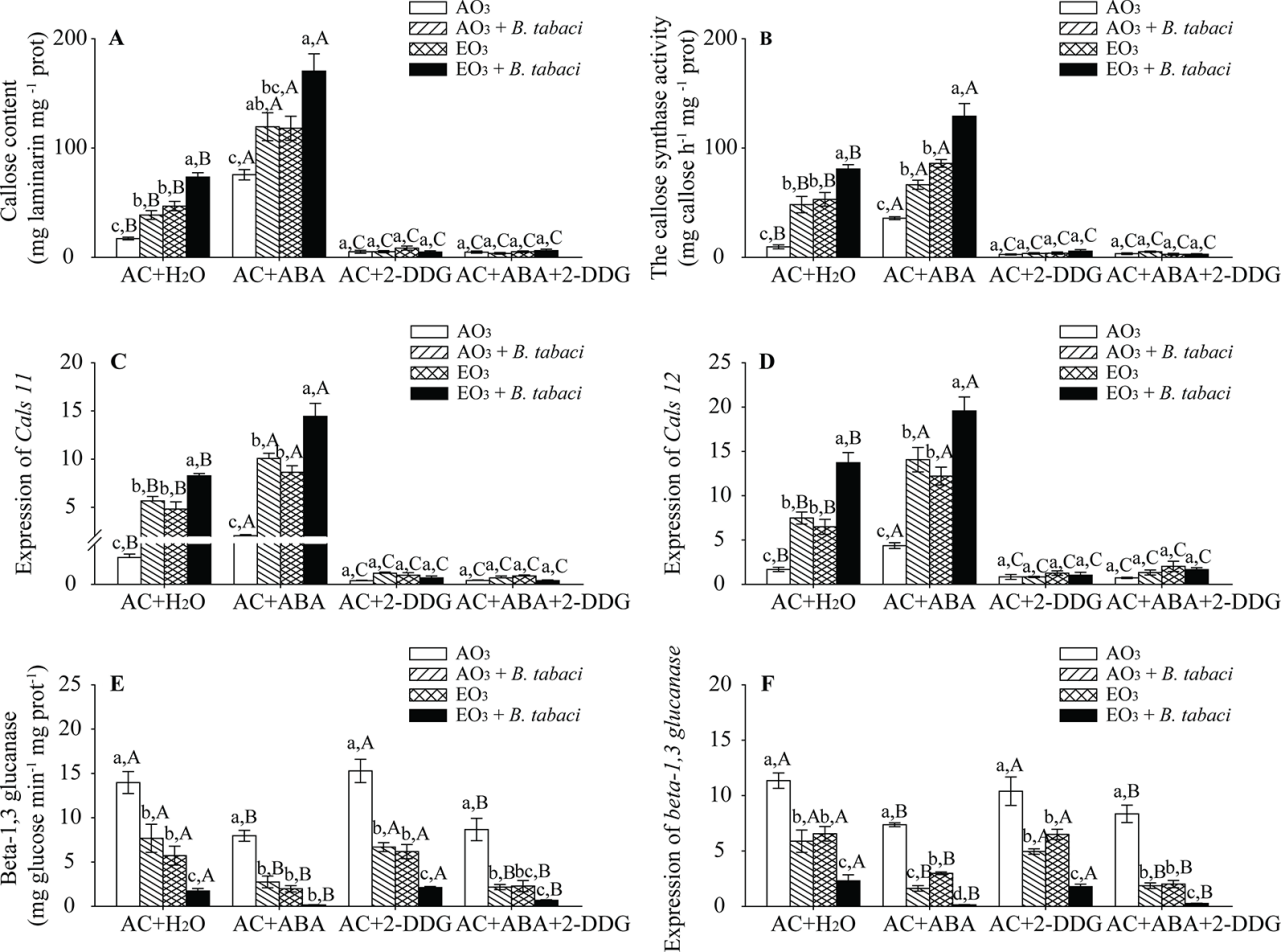
Callose content, enzyme activity, and fold-change in the expression of related genes involved in the callose synthesis and degradation for wild-type AC plants treated with H_2_O, ABA, 2-DGG, and ABA plus 2-DDG, grown under ambient O_3_ (AO_3_) and elevated O_3_ (EO_3_) with and without *B. tabaci* infestation. **(A)** Callose content, **(B)** callose synthase activity, **(C)** relative expression of *Cals 11* gene, **(D)** relative expression of *Cals 12* gene, **(E)** beta-1,3 glucanase activity, and **(F)** relative expression of beta-1,3 glucanase gene. Each value represents the average (± SE) of four OTCs (eight plants for each treatment per OTC). Different lowercase letters indicate significant differences among the combinations of *B. tabaci* infestation and O_3_ concentrations within the same chemical reagent treatment. Different uppercase letters indicate significant differences among different chemical reagent treatments within the same O_3_ concentrations and *B. tabaci* infestation, as determined by Tukey's multiple range test at *P* < 0.05.

Our results showed that, regardless of O_3_ concentration and *B. tabaci* infestation, the foliar callose content was significantly higher in the AC/ABA plants than in the AC/H_2_O plants but was obviously lower in the AC/2-DDG plants and the AC/ABA/2-DDG plants than the AC/H_2_O plants. Elevated O_3_ increased the callose content by nearly 3-fold in the AC/H_2_O plants without *B. tabaci* infestation and by 2-fold in the AC/ABA plants without *B. tabaci* infestation. With *B. tabaci* infestation and ambient O_3_, the content of foliar callose was increased by 1.2-fold in the AC/H_2_O plants and by 0.6-fold in the AC/ABA plants. With *B. tabaci* infestation and elevated O_3_, foliar callose content was improved by 3.3-fold in the AC/H_2_O plants and by 1.2-fold in the AC/ABA plants. However, elevated O_3_ and *B. tabaci* infestation, individually and combined, had little effect on the content of foliar callose in the AC/2-DDG plants and AC/ABA/2-DDG plants. Furthermore, the enzyme activity of callose synthase and the relative expression of key genes (*Cals11* and *Cals12* genes) were consistent with the content of foliar callose, as they were increased by elevated O_3_ and *B. tabaci* infestation individually and together in the AC/H_2_O plants and the AC/ABA plants, while they were not affected in the AC/2-DGG plants and AC/ABA/2-DDG plants (**Figures 6A–D**, **Figure S.2**; **Table S.5**). Moreover, we also found that *B. tabaci* infestation and elevated O_3_, individually and combined, decreased the enzyme activity and gene expression of beta-1,3 glucanase in different treatments (**Figures 6E, F**; **Table S.5**).

**Figure 7:**
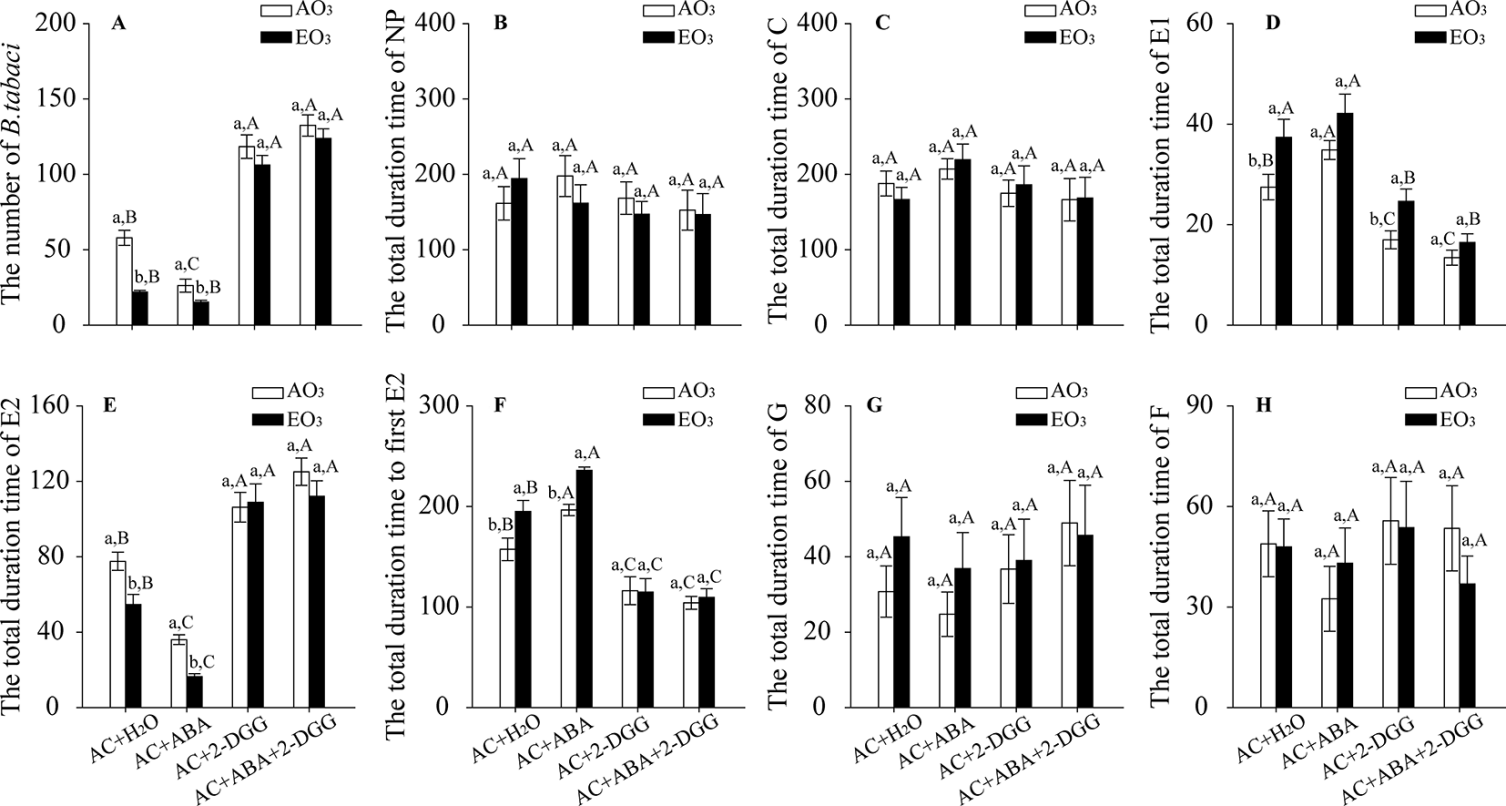
Population abundance and feeding behavior of *B. tabaci* when fed on wild-type AC plants treated with H_2_O, ABA, 2-DGG, and ABA plus 2-DDG, grown under ambient O_3_ (AO_3_) and elevated O_3_ (EO_3_) with and without *B. tabaci* infestation. **(A)** Population abundance (number per plant). Each value represents the average (± SE) of four OTCs (12 plants for each genotype per OTC). **(B)** ‘Nonpenetration' (NP, stylets are outside the plants), **(C)** ‘pathway' (C, mostly intramural probing activities between mesophyll or parenchyma cells), **(D)** ‘salivation' (E1, salivary secretion into sieve elements), **(E)** ‘phloem ingestion' (E2, ingesting the phloem sap), **(F)** total time to first E2, **(G)** ‘xylem ingestion' (G, stylet penetration of tracheary elements), and (H) derailed stylets (F, stylets are exhibiting penetration difficulties). Values are the mean (± SE) of 15 biological replicates. Different lowercase letters indicate significant differences between ambient O_3_ and elevated O_3_ within the same chemical reagent treatment. Different uppercase letters indicate significant differences among different chemical reagent treatments within the same O_3_ treatment, as determined by Tukey's multiple range test at *P* < 0.05.

O_3_ exposure decreased the population abundance of *B. tabaci* by 62% in the AC/H_2_O plants and by 42% in the AC/ABA plants. However, elevated O_3_ had little effect on the population abundance of *B. tabaci* associated with the AC/2-DDG and AC/ABA/2-DDG plants. The population abundance of *B. tabaci* significantly decreased in the AC/ABA plants compared with the AC/H_2_O plants but increased in the AC/2-DDG plants and AC/ABA/2-DDG plants regardless of O_3_ levels (**Figure 7A**). For the feeding behavior of *B. tabaci*, elevated O_3_ increased the total duration of E1 by 36%, decreased the total duration of E2 by 29%, and prolonged the total time to the first E2 by 24% in the AC/H_2_O plants. In AC/ABA plants, elevated O_3_ increased the total duration of E1 by 21%, decreased the total duration of E2 by 54%, and prolonged the total time to the first E2 by 20%. However, it had little effect on the total duration of E1, the total duration of E2, and the total time to first E2 in the AC/2-DGG and AC/ABA/2-DDG plants. Furthermore, regardless of O_3_ level, the *B. tabaci* had a longer E1 phase and total time to the first E2, but a shorter E2 phase on the AC/ABA plants than on the AC/H_2_O plants. In contrast, compared with AC/H_2_O plants, the *B. tabaci* associated with the AC/2-DDG and AC/ABA/2-DDG plants had a shorter E1 phase and total time to the first E2, but a longer E2 phase (**Figures 7B–H**; **Table S.6**).

## Discussion

Greenhouse gases, such as elevated CO_2_ or O_3_, can modulate phytohormone-dependent signals in plants (Tamaoki, 2008; Pellegrini et al., 2016), and such priming defenses have significant consequences for the performance of herbivorous insects (Robinson et al., 2012; Zavala et al., 2013). In this study, we reported that elevated O_3_ induced callose deposition in tomato plants and therefore enhanced plant resistance to *B. tabaci* in terms of reducing feeding efficiency and population abundance. This cascading effect was ABA-dependent. Several studies have indicated that phloem-sucking insects tend to upregulate the ABA signaling pathway in some plant species, which is considered a decoy strategy to facilitate their colonization by suppressing JA defenses (Studham and MacIntosh, 2013; Hillwig et al., 2016). Our study, however, demonstrated that the activation of ABA signaling can induce callose deposition, conferring an effective defense against whitefly infestation.

The activation of the ABA signaling pathway is widely regarded as an important characteristic of plant response to drought stress (Cutler et al., 2010; Osakabe et al., 2014). Recent studies demonstrated that O_3_ exposure can activate the ABA signaling pathway, with a significant increase in ABA content and the expression of ABA-related genes (Cotrozzi et al., 2017; McAdam et al., 2017; Landi et al., 2019). Furthermore, the upregulation of the *NCED* gene, which encodes a rate-limiting enzyme (nine-cis carotenoid cleavage dioxygenase) in the ABA synthesis pathway, was responsible for the augmentation of ABA levels in Arabidopsis leaves under a vapor pressure deficit (McAdam and Brodribb, 2016). Our results also found that the increase in foliar ABA content induced by O_3_ exposure was absent in the *not* plants but not in the AC plants, and, since the *not* plants have a mutation in the *NCED1* gene, this suggests that the *NCED* genes are necessary for O_3_-induced foliar ABA accumulation. Moreover, the activation of the ABA signaling pathway typically triggers the movement of leaf stomata, which are involved in avoiding the negative effects of O_3_ exposure on host plants (Feng et al., 2012; Merilo et al., 2013; Feng et al., 2018). Similarly, in our study, elevated O_3_ activated the ABA signaling pathway and decreased stomatal conductance and increased stomatal closure in the AC plants. However, the ABA-deficient *not* plants with a deficiency in closing stomata upon O_3_ exposure were highly sensitive to elevated O_3_. Notably, increased O_3_-induced leaf injury indicated that ABA signaling was involved in plant tolerance to O_3_ through stomatal regulation.

Elevated O_3_ can prime tomato leaves for an enhanced hormone-dependent defense against subsequent herbivorous insect infestations. We found that elevated O_3_ activated the SA signaling pathway, with a significant increase in the SA content and the expression of downstream defense genes (*PR*). Although *B. tabaci* infestation activated the SA signaling pathway, recent studies suggested that the JA signaling pathway was more effective than the SA pathway in resistance against B. tabaci infestation (Li et al., 2014; Xu et al., 2019). For example, in Arabidopsis, the development of *B. tabaci* nymphs is delayed on JA-activated *cev1* plants and on SA-deficient *npr1* plants. Furthermore, *B.tabaci* feeding on *npr1* plants with MeJA treatment showed delayed development of nymphs relative to solvent (0.001% ethanol)-treated *npr1* plants (Zarate et al., 2007). Our results showed that elevated O_3_ had little effect on the JA signaling pathway in tomato plants with and without *B. tabaci* infestation. The current study suggested that the SA and JA signaling pathways were not involved in the negative effects of elevated O_3_ on *B. tabaci*. Except for the SA and JA signaling pathways, ABA signaling is also critical for regulating plant responses to infestations by herbivorous insects. Infestation by phloem-sucking insects can increase the ABA content and the expression of ABA signaling-related genes in their host plants (Quintana-Camargo et al., 2015; Hillwig et al., 2016). Furthermore, the importance of ABA signaling in plant resistance to insects has been attributed to its role in inducing foliar callose deposition (Liu et al., 2017). Our results showed that *not* plants with little callose accumulation had an increased *B. tabaci* population abundance and phloem-feeding efficiency, while ABA-treated plants with increased callose accumulation maintained a reduced *B. tabaci* population abundance and phloem-feeding efficiency. Furthermore, when ABA signaling was activated but downstream callose accumulation was inhibited by 2-DGG, the negative effects of ABA signaling on the performance of *B. tabaci* disappeared, suggesting that ABA signaling enhanced plant resistance to *B. tabaci* by inducing increased callose deposition. The ABA signaling pathway improved callose accumulation by inhibiting the transcription of the callose degradation enzyme beta-1,3-glucanase (Oide et al., 2013). Callose accumulation is dependent on not only the hydrolyzing enzyme beta-1,3-glucanase but also callose synthase, which catalyzes the synthesis of callose in response to biotic and abiotic stresses (Verma and Hong, 2001). In Arabidopsis, the accumulation of foliar callose was significantly lower in callose synthase-deficient *pmr4-1* plants with a mutation in the callose synthase gene *AtGSL5* than in wild-type Col-0 plants under 80-µM ABA treatment, which indicated that ABA-induced callose accumulation depended on callose synthase (Flors et al., 2008). Our results found that the *not* plants had higher enzyme activity and gene expression of beta-1,3-glucanase, lower enzyme activity and transcripts of callose synthase, and less foliar callose content than AC plants. Moreover, ABA-treated plants had decreased enzyme activity and gene expression of beta-1,3-glucanase but increased enzyme activity and gene expression of callose synthase and foliar callose contents. This is consistent with a previous study showing that a decrease in the hydrolyzing enzyme and an increase in callose synthase resulted in an enhancement of callose accumulation in exogenous ABA-treated rice, which shortened the duration of phloem ingestion of brown planthopper (Liu et al., 2017). Thus, the activation of the ABA signaling pathway increased callose synthase but suppressed callose degradation to accumulate callose in plant tissue, which decreased the phloem feeding of *B. tabaci*.

Callose deposition is a ubiquitous phloem-based defensive mechanism that is employed in many plant species to resist attacks by phloem-sucking insects with stylet-like mouthparts that feed mainly on phloem sap (Li et al., 2017; Bak et al., 2017). In contrast to chemically induced defenses, callose deposition on the sieve plates, leading to sieve occlusion, serves as a physical barrier to prevent phloem-sucking insects from ingesting the flow of phloem sap (Fu et al., 2014; Zhai et al., 2017). For example, infestation by brown planthoppers induced the expression of callose synthase genes and callose accumulation, resulting in a decreased duration of phloem ingestion (Hao et al., 2008). Our results showed that the total phloem-feeding time was significantly prolonged in association with the exogenous application of 2-DDG on the AC plants. Furthermore, the negative effects of elevated O_3_ on the phloem-feeding behavior of *B. tabaci* was also absent in the 2-DDG-treated AC plants, which suggested that callose deposition contributed to O_3_-induced priming defense against *B. tabaci*. This is consistent with previous studies showing that preliminary reagent (e.g., indole-3-carboxylic acid and β‐amino‐butyric acid) treatment-induced or abiotic stress (e.g., high silicon concentration)-induced defense priming against subsequent insects or pathogen challenge is dependent on callose accumulation (Baccelli and Mauch-Mani, 2016; Yang et al., 2018; Avramova, 2019). In Arabidopsis, indole-3-carboxylic acid- and β‐amino‐butyric acid-induced callose priming against *P. cucumerina* infection is blocked in ABA-deficient mutants such *as npq2, aba1‐5*, and *aba2.3* (Ton and Mauch-Mani, 2004; Gamir et al., 2018). Likewise, elevated O_3_ had little effect on the content of foliar callose and the population abundance and phloem-feeding behavior of *B. tabaci* in the ABA-deficient *not* plants, indicating that the ABA signaling pathway was required for callose-mediated priming defense.

In conclusion, our results revealed that elevated O_3_ activated the ABA signaling pathway and induced the deposition of callose, which is a disadvantage for the feeding efficiency and population fitness of *B. tabaci* associated with tomato plants. This study has generated several significant findings. First, stomatal closure dependent on the ABA signaling pathway enhanced the tolerance of tomato plants to O_3_ exposure. Second, ABA-induced callose accumulation reduced the fitness of *B. tabaci* on tomato plants under elevated O_3_. Finally, our results suggest that tomato plants may suffer less *B. tabaci* damage under elevated O_3_ environments due to an O_3_-induced priming defense. Further research is needed to elucidate the regulation of callose synthase genes in response to the ABA signaling pathway and the early events upstream of the ABA signaling pathway following O_3_-induced priming defense. Given that increasing atmospheric O_3_ and other environmental stresses (such as atmospheric CO_2_, drought, and UV) always occur together, more research is needed to further investigate the interactive impacts of multiple environmental stresses on *B. tabaci* performance and the function of ABA signals in regulating these interactive effects on pest insect performance.

## Data Availability Statement

The raw data supporting the conclusions of this article will be made available by the authors, without undue reservation, to any qualified researcher.

## Author Contributions

HG, YS, and FG planned and designed the research. HG performed experiments, conducted fieldwork, and analyzed data. CL provided tomato seeds. HY provided field support. HG wrote the first draft of the manuscript, and YS and FG contributed to the subsequent manuscript development.

## Funding

This project was supported by the National Key Research and Development Plan (2017YFD0200400) and the National Natural Science Foundation of China (no.31572059).

## Conflict of Interest

The authors declare that the research was conducted in the absence of any commercial or financial relationships that could be construed as a potential conflict of interest.

## References

[B1] Ainsworth E. A. Yendrek C. R. Sitch S. Collins W. J. Emberson L. D. (2012). The effects of tropospheric ozone on net primary productivity and implications for climate change. Annu. Rev. Plant Biol. 63, 637–661. 10.1146/annurev-arplant-042110-103829 22404461

[B2] Ashmore M. R. (2005). Assessing the future global impacts of ozone on vegetation. Plant Cell Environ. 28 (8), 949–964. 10.1111/j.1365-3040.2005.01341.x

[B3] Asselbergh B. Höfte M. (2007). Basal tomato defences to *Botrytis cinerea* include abscisic acid-dependent callose formation. Physiol. Mol. Plant Pathol. 71 (1-3), 33–40. 10.1016/j.pmpp.2007.10.001

[B4] Avramova Z. (2019). Defence-related priming and responses to recurring drought: Two manifestations of plant transcriptional memory mediated by the ABA and JA signalling pathways. Plant Cell Environ. 42 (3), 983–997. 10.1111/pce.13458 30299553

[B5] Baccelli I. Mauch-Mani B. (2016). Beta-aminobutyric acid priming of plant defense: the role of ABA and other hormones. Plant Mol. Biol. 91 (6), 703–711. 10.1007/s11103-015-0406-y 26584561

[B6] Bak A. Cheung A. L. Yang C. Whitham S. A. Casteel C. L. (2017). A viral protease relocalizes in the presence of the vector to promote vector performance. Nat. Commun. 8, 14493. 10.1038/ncomms14493 28205516 PMC5316897

[B7] Cheng X. Zhu L. He G. (2013). Towards understanding of molecular interactions between rice and the brown planthopper. Mol. Plant 6 (3), 621–634. 10.1093/mp/sst030 23396040

[B8] Cooper O. R. Parrish D. D. Ziemke J. Cupeiro M. Galbally I. E. Gilge S. (2014). Global distribution and trends of tropospheric ozone: An observation-based review. Elem. Sci. Anth. 2, 29 10.12952/journal.elementa.000029

[B9] Cotrozzi L. Pellegrini E. Guidi L. Landi M. Lorenzini G. Massai R. (2017). Losing the warning signal: drought compromises the cross-talk of signaling molecules in Quercus ilex exposed to ozone. Front. Plant Sci. 8, 1020. 10.3389/fpls.2017.01020 28674543 PMC5475409

[B10] Cui H. Y. Sun Y. C. Su J. W. Ren Q. Li C. Y. Ge F. (2012). Elevated O_3_ reduces the fitness of *Bemisia tabaci via* enhancement of the SA-dependent defense of the tomato plant. Arthropod-Plant Inte. 6 (3), 425–437. 10.1007/s11829-012-9189-0

[B11] Cui H. Su J. Wei J. Hu Y. Ge F. (2014). Elevated O_3_ enhances the attraction of whitefly-infested tomato plants to *Encarsia formosa* . Sci. Rep. 4, 5350. 10.1038/srep05350 24939561 PMC4061550

[B12] Cutler S. R. Rodriguez P. L. Finkelstein R. R. Abrams S. R. (2010). Abscisic acid: emergence of a core signaling network. Annu. Rev. Plant Biol. 61, 651–679. 10.1146/annurev-arplant-042809-112122 20192755

[B13] Dalton R. (2006). Whitefly infestations: the Christmas invasion. Nature 443, 898–900. 10.1038/443898a 17066003

[B14] De Barro P. J. Liu S. S. Boykin L. M. Dinsdale A. B. (2011). *Bemisia tabaci*: a statement of species status. Annu. Rev. Entomol. 56, 1–19. 10.1146/annurev-ento-112408-085504 20690829

[B15] Du M. Zhai Q. Deng L. Li S. Li H. Yan L. (2014). Closely related NAC transcription factors of tomato differentially regulate stomatal closure and reopening during pathogen attack. Plant Cell 26 (7), 3167–3184. 10.1105/tpc.114.128272 25005917 PMC4145139

[B16] Expósito-Rodríguez M. Borges A. A. Borges-Pérez A. Pérez J. A. (2008). Selection of internal control genes for quantitative real-time RT-PCR studies during tomato development process. BMC Plant Biol. 8 (1), 131. 10.1186/1471-2229-8-131 19102748 PMC2629474

[B17] Feng Z. Tang H. Uddling J. Pleijel H. Kobayashi K. Zhu J. (2012). A stomatal ozone flux–response relationship to assess ozone-induced yield loss of winter wheat in subtropical China. Environ. Pollut. 164, 16–23. 10.1016/j.envpol.2012.01.014 22310057

[B18] Feng Z. Büker P. Pleijel H. Emberson L. Karlsson P. E. Uddling J. (2018). A unifying explanation for variation in ozone sensitivity among woody plants. Global Change Biol. 24 (1), 78–84. 10.1111/gcb.13824 28722164

[B19] Flors V. Ton J. Jakab G. Mauch-Mani B. (2005). Abscisic acid and callose: team players in defence against pathogens? J. Phytopathol. 153 (7-8), 377–383. 10.1111/j.1439-0434.2005.00987.x

[B20] Flors V. Ton J. van Doorn R. Jakab G. Garcia-Agustin P. Mauch-Mani B. (2008). Interplay between JA SA and ABA signalling during basal and induced resistance against *Pseudomonas syringae* and *Alternaria brassicicola*. Plant J. 54 (1), 81–92. 18088307 10.1111/j.1365-313X.2007.03397.x

[B21] Fu M. Xu M. Zhou T. Wang D. Tian S. Han L. (2014). Transgenic expression of a functional fragment of harpin protein Hpa1 in wheat induces the phloem-based defence against English grain aphid. J. Exp. Bot. 65 (6), 1439–1453. 10.1093/jxb/ert488 24676030 PMC3967084

[B22] Gamir J. Pastor V. Sánchez-Bel P. Agut B. Mateu D. García-Andrade J. (2018). Starch degradation, abscisic acid and vesicular trafficking are important elements in callose priming by indole-3-carboxylic acid in response to *Plectosphaerella cucumerina* infection. Plant J. 96 (3), 518–531. 10.1111/tpj.14045 30051514

[B23] García-Andrade J. Ramírez V. Flors V. Vera P. (2011). Arabidopsis *ocp_3_* mutant reveals a mechanism linking ABA and JA to pathogen-induced callose deposition. Plant J. 67 (5), 783–794. 10.1111/j.1365-313X.2011.04633.x 21564353

[B24] Guo H. Sun Y. Peng X. Wang Q. Harris M. Ge F. (2015). Up-regulation of abscisic acid signaling pathway facilitates aphid xylem absorption and osmoregulation under drought stress. J. Exp. Bot. 67 (3), 681–693. 10.1093/jxb/erv481 26546578 PMC4737068

[B25] Guo H. Sun Y. Yan H. Li C. Ge F. (2018). O_3_-induced leaf senescence in tomato plants is ethylene signaling-dependent and enhances the population abundance of *Bemisia tabaci* . Front. Plant Sci. 9, 764. 10.3389/fpls.2018.00764 29946327 PMC6005859

[B26] Gupta P. Duplessis S. White H. Karnosky D. F. Martin F. Podila G. K. (2005). Gene expression patterns of trembling aspen trees following long-term exposure to interacting elevated CO_2_ and tropospheric O_3_ . New Phytol. 167 (1), 129–142. 10.1111/j.1469-8137.2005.01422.x 15948836

[B27] Hao P. Liu C. Wang Y. Chen R. Tang M. Du B. (2008). Herbivore-induced callose deposition on the sieve plates of rice: an important mechanism for host resistance. Plant Physiol. 146 (4), 1810–1820. 10.1104/pp.107.111484 18245456 PMC2287352

[B28] Hilker M. Schmülling T. (2019). Stress priming, memory, and signalling in plants. Plant Cell Environ. 42 (3), 753–761. 10.1111/pce.13526 30779228

[B29] Hillwig M. S. Chiozza M. Casteel C. L. Lau S. T. Hohenstein J. Hernández E. (2016). Abscisic acid deficiency increases defence responses against *Myzus persicae* in *Arabidopsis* . Mol. Plant Pathol. 17 (2), 225–235. 10.1111/mpp.12274 25943308 PMC6638517

[B30] IPCC (2013). Intergovernmental Panel on Climate Change Website. Available at: www.ipcc.ch [accessed August 12, 2017].

[B31] Kerchev P. I. Karpińska B. Morris J. Hussain A. Verrall S. R. Hedley P. E. (2013). Vitamin C and the abscisic acid-insensitive 4 transcription factor are important determinants of aphid resistance in Arabidopsis. Antioxid. Redox Signal. 18 (16), 2091–2105. 10.1089/ars.2012.5097 23343093

[B32] Landi M. Cotrozzi L. Pellegrini E. Remorini D. Tonelli M. Trivellini A. (2019). When “thirsty” means “less able to activate the signaling wave trigged by a pulse of ozone”: A case of study in two Mediterranean deciduous oak species with different drought sensitivity. Sci. Total Environ. 657, 379–390. 10.1016/j.scitotenv.2018.12.012 30550902

[B33] Li X. M. Zhang L. H. Ma L. J. Li Y. Y. (2011). Elevated Carbon Dioxide and/or Ozone Concentrations Induce Hormonal Changes in *Pinus tabulaeformis* . J. Chem. Ecol. 37, 779–784. 10.1007/s10886-011-9975-7 21611809

[B34] Li R. Weldegergis B. T. Li J. Jung C. Qu J. Sun Y. (2014). Virulence factors of geminivirus interact with *MYC2* to subvert plant resistance and promote vector performance. Plant Cell 26, 4991–5008. 10.1105/tpc.114.133181 25490915 PMC4311212

[B35] Li P. Shu Y. N. Fu S. Liu Y. Q. Zhou X. P. Liu S. S. (2017). Vector and nonvector insect feeding reduces subsequent plant susceptibility to virus transmission. New Phytol. 215 (2), 699–710. 10.1111/nph.14550 28382644

[B36] Liu B. Preisser E. L. Chu D. Pan H. Xie W. Wang S. (2013). Multiple forms of vector manipulation by a plant-infecting virus: *Bemisia tabaci* and tomato yellow leaf curl virus. J. Virol. 87 (9), 4929–4937. 10.1128/JVI.03571-12 23408638 PMC3624301

[B37] Liu J. Du H. Ding X. Zhou Y. Xie P. Wu J. (2017). Mechanisms of callose deposition in rice regulated by exogenous abscisic acid and its involvement in rice resistance to *Nilaparvata lugens Stål* (Hemiptera: Delphacidae). Pest Manage. Sci. 73 (12), 2559–2568. 10.1002/ps.4655 28664567

[B38] Luna E. Pastor V. Robert J. Flors V. Mauch-Mani B. Ton J. (2011). Callose deposition: a multifaceted plant defense response. Mol. Plant-Microbe In. 24 (2), 183–193. 10.1094/MPMI-07-10-0149 20955078

[B39] Mauch-Mani B. Baccelli I. Luna E. Flors V. (2017). Defense priming: an adaptive part of induced resistance. Annu. Rev. Plant Biol. 68, 485–512. 10.1146/annurev-arplant-042916-041132 28226238

[B40] McAdam S. A. Brodribb T. J. (2016). Linking turgor with ABA biosynthesis: implications for stomatal responses to vapor pressure deficit across land plants. Plant Physiol. 171 (3), 2008–2016. 10.1104/pp.16.00380 27208264 PMC4936570

[B41] McAdam E. L. Brodribb T. J. McAdam S. A. (2017). Does ozone increase ABA levels by non-enzymatic synthesis causing stomata to close? Plant Cell Environ. 40 (5), 741–747. 10.1111/pce.12893 28042679

[B42] Merilo E. Laanemets K. Hu H. Xue S. Jakobson L. Tulva I. (2013). PYR/RCAR receptors contribute to ozone-, reduced air humidity-, darkness-, and CO_2_-induced stomatal regulation. Plant Physiol. 162 (3), 1652–1668. 10.1104/pp.113.220608 23703845 PMC3707544

[B43] Ohara T. A. H. K. Akimoto H. Kurokawa J. I. Horii N. Yamaji K. Yan X. (2007). An Asian emission inventory of anthropogenic emission sources for the period 1980–2020. Atmos. Chem. Phys. 7 (16), 4419–4444. 10.5194/acp-7-4419-2007

[B44] Oide S. Bejai S. Staal J. Guan N. Kaliff M. Dixelius C. (2013). A novel role of *PR2* in abscisic acid (ABA) mediated, pathogen-induced callose deposition in *Arabidopsis thaliana* . New Phytol. 200 (4), 1187–1199. 10.1111/nph.12436 23952213

[B45] Osakabe Y. Yamaguchi-Shinozaki K. Shinozaki K. Tran L. S. P. (2014). ABA control of plant macroelement membrane transport systems in response to water deficit and high salinity. New Phytol. 202 (1), 35–49. 10.1111/nph.12613 24283512

[B46] Pellegrini E. Trivellini A. Cotrozzi L. Vernieri P. Nali C. (2016). “Involvement of phytohormones in plant responses to ozone,” in Plant Hormones under Challenging Environmental Factors. Eds. Ahammed G. Yu J. Q. (Dordrecht: Springer Press), 215–245. 10.1007/978-94-017-7758-2_9

[B47] Peltonen P. A. Vapaavuori E. Heinonen J. Julkunen-tiitto R. Holopainen J. K. (2010). Do elevated atmospheric CO_2_ and O_3_ affect food quality and performance of folivorous insects on silver birch? Global Change Biol. 16 (3), 918–935. 10.1111/j.1365-2486.2009.02073.x

[B48] Quintana-Camargo M. Méndez-Morán L. Ramirez-Romero R. Gurrola-Díaz C. M. Carapia-Ruiz V. Ibarra-Laclette E. (2015). Identification of genes differentially expressed in husk tomato (Physalis philadelphica) in response to whitefly (Trialeurodes vaporariorum) infestation. Acta Physiol. Plant 37 (2), 29. 10.1007/s11738-015-1777-z

[B49] Robinson E. A. Ryan G. D. Newman J. A. (2012). A meta-analytical review of the effects of elevated CO_2_ on plant–arthropod interactions highlights the importance of interacting environmental and biological variables. New Phytol. 194 (2), 321–336. 10.1111/j.1469-8137.2012.04074.x 22380757

[B50] Studham M. E. MacIntosh G. C. (2013). Multiple phytohormone signals control the transcriptional response to soybean aphid infestation in susceptible and resistant soybean plants. Mol. Plant-Microbe In. 26 (1), 116–129. 10.1094/MPMI-05-12-0124-FI 22992001

[B51] Tamaoki M. (2008). The role of phytohormone signaling in ozone-induced cell death in plants. Plant Signal. Behav. 3 (3), 166–174. 10.4161/psb.3.3.5538 19513211 PMC2634110

[B52] Tan X. L. Chen J. L. Benelli G. Desneux N. Yang X. Q. Liu T. X. (2017). Pre-infestation of tomato plants by aphids modulates transmission-acquisition relationship among whiteflies, tomato yellow leaf curl virus (TYLCV) and plants. Front. Plant Sci. 8, 1597. 10.3389/fpls.2017.01597 29018457 PMC5614976

[B53] Thompson A. J. Thorne E. T. Burbidge A. Jackson A. C. Sharp R. E. Taylor I. B. (2004). Complementation of *notabilis*, an abscisic acid-deficient mutant of tomato: importance of sequence context and utility of partial complementation. Plant Cell Environ. 27 (4), 459–471. 10.1111/j.1365-3040.2003.01164.x

[B54] Ton J. Mauch-Mani B. (2004). β-amino-butyric acid-induced resistance against necrotrophic pathogens is based on ABA-dependent priming for callose. Plant J. 38 (1), 119–130. 10.1111/j.1365-313X.2004.02028.x 15053765

[B55] Verma D. P. S. Hong Z. (2001). Plant callose synthase complexes. Plant Mol. Biol. 47 (6), 693–701. 10.1023/A:1013679111111 11785931

[B56] Xiao D. Duan X. Zhang M. Sun T. Sun X. Li F. (2018). Changes in nitric oxide levels and their relationship with callose deposition during the interaction between soybean and Soybean mosaic virus. Plant Biol. 20 (2), 318–326. 10.1111/plb.12663 29125664

[B57] Xu H. X. Qian L. X. Wang X. W. Shao R. X. Hong Y. Liu S. S. (2019). A salivary effector enables whitefly to feed on host plants by eliciting salicylic acid-signaling pathway. PNAS 116 (2), 490–495. 10.1073/pnas.1714990116 30584091 PMC6329982

[B58] Yang L. Li P. Li F. Ali S. Sun X. Hou M. (2018). Silicon amendment to rice plants contributes to reduced feeding in a phloem-sucking insect through modulation of callose deposition. Ecol. Evol. 8 (1), 631–637. 10.1002/ece3.3653 29321899 PMC5756854

[B59] Yao L. Zhong Y. Wang B. Yan J. Wu T. (2019). BABA application improves soybean resistance to aphid through activation of phenylpropanoid metabolism and callose deposition. Pest Manag. Sci. 76 (1), 384–394. 10.1002/ps.5526 31222925

[B60] Zarate S. I. Kempema L. A. Walling L. L. (2007). Silverleaf whitefly induces salicylic acid defenses and suppresses effectual jasmonic acid defenses. Plant Physiol. 143, 866–875. 10.1104/pp.106.090035 17189328 PMC1803729

[B61] Zavala J. A. Nabity P. D. DeLucia E. H. (2013). An emerging understanding of mechanisms governing insect herbivory under elevated CO_2_ . Annu. Rev. Entomol. 58, 79–97. 10.1146/annurev-ento-120811-153544 22974069

[B62] Zhai Y. Li P. Mei Y. Chen M. Chen X. Xu H. (2017). Three MYB genes co-regulate the phloem-based defence against English grain aphid in wheat. J. Exp. Bot. 68 (15), 4153–4169. 10.1093/jxb/erx204 28922762

[B63] Zhang H. Shi W. L. You J. F. Bian M. D. Qin X. M. Yu H. (2015). Transgenic Arabidopsis thaliana plants expressing a β-1, 3-glucanase from sweet sorghum (Sorghum bicolor L.) show reduced callose deposition and increased tolerance to aluminium toxicity. Plant Cell Environ. 38 (6), 1178–1188. 10.1111/pce.12472 25311645

