# Supplementary material for: O3-Induced Priming Defense Associated With the Abscisic Acid Signaling Pathway Enhances Plant Resistance to Bemisia tabaci
Source: Front Plant Sci. 2020 Feb 26;11:93. doi: 10.3389/fpls.2020.00093 (PMC7069499; doi:10.3389/fpls.2020.00093)

Supplementary material

Table S.1 Population abundance and feeding behavior of *B. tabaci* when fed on wild-type AC and ABA-deficient mutants *not* grown under ambient O_3_ and elevated O_3_.

| Variable | O_3_ | | Plant genotype | | O_3_ 🞨 Plant genotype | |
| --- | --- | --- | --- | --- | --- | --- |
|  | *F* | *P* | *F* | *P* | *F* | *P* |
| The number of *B.tabaci* | 11.603 | 0.014 | 11.472 | 0.001 | 2.379 | 0.129 |
| The total duration of NP | 0.211 | 0.648 | 1.072 | 0.305 | 0.102 | 0.750 |
| The total duration of C | 0.758 | 0.388 | 0.959 | 0.332 | 0.280 | 0.599 |
| The total duration of E1 | 8.045 | 0.006 | 14.820 | < 0.001 | 1.358 | 0.249 |
| The total duration of E2 | 7.205 | 0.010 | 27.508 | < 0.001 | 0.522 | 0.473 |
| The total duration time to first E2 | 15.049 | < 0.001 | 70.068 | < 0.001 | 3.706 | 0.059 |
| The total duration of G | 0.079 | 0.779 | 13.223 | 0.001 | 1.274 | 0.264 |
| The total duration of F | 0.094 | 0.761 | 2.495 | 0.120 | 1.234 | 0.271 |

Table S.2 Growth traits of two tomato genotypes (AC and *not*) grown under ambient O_3_ and elevated O_3_ without *B. tabaci* infestation.

| No. | Variable | Source | F | P |
| --- | --- | --- | --- | --- |
| 1 | Photosynthetic rate | O_3_ | 739.078 | < 0.001 |
|  |  | Plant genotype | 19.260 | < 0.001 |
|  |  | O_3_🞨 Plant genotype | 0.369 | 0.544 |
| 2 | Biomass | O_3_ | 320.694 | < 0.001 |
|  |  | Plant genotype | 105.390 | < 0.001 |
|  |  | O_3_🞨 Plant genotype | 0.481 | 0.488 |
| 3 | stippled leaves | Ozone | 148.795 | 0.001 |
|  |  | Plant genotype | 52.350 | < 0.001 |
|  |  | O_3_🞨 Plant genotype | 5.500 | 0.021 |
| 4 | burned leaves | O_3_ | 65.165 | 0.003 |
|  |  | Plant genotype | 1.155 | 0.287 |
|  |  | O_3_🞨 Plant genotype | 1.240 | 0.270 |
| 5 | curl leaves | O_3_ | 112.191 | < 0.001 |
|  |  | Plant genotype | 13.944 | < 0.001 |
|  |  | O_3_🞨 Plant genotype | 15.375 | < 0.001 |
| 6 | deciduous leaves | O_3_ | 61.084 | 0.004 |
|  |  | Plant genotype | 13.009 | 0.001 |
|  |  | O_3_🞨 Plant genotype | 9.448 | 0.003 |
| 7 | ROS | O_3_ | 41.247 | < 0.001 |
|  |  | Plant genotype | 3.073 | 0.118 |
|  |  | O_3_🞨 Plant genotype | 5.375 | 0.081 |
| 8 | stomatal conductance | O_3_ | 61.55 | < 0.001 |
|  |  | Plant genotype | 77.953 | < 0.001 |
|  |  | O_3_🞨 Plant genotype | 50.223 | < 0.001 |
| 9 | rate of closed stomata | O_3_ | 6.868 | 0.034 |
|  |  | Plant genotype | 45.41 | < 0.001 |
|  |  | O_3_🞨 Plant genotype | 11.049 | 0.029 |

Table S.3 ABA content and relative expression of related genes involved in the ABA signaling pathway for two tomato genotypes grown under ambient O_3_ and elevated O_3_ with *B. tabaci* infestation.

| Variable | O_3_ | | Plant genotype | | *B. tabaci* infestation | | O_3_ 🞨 Plant genotype | | O_3_ 🞨  *B. tabaci* infestation | | *B. tabaci* infestation 🞨 Plant genotype | | O_3_ 🞨 Plant genotype 🞨*B. tabaci* infestation | |
| --- | --- | --- | --- | --- | --- | --- | --- | --- | --- | --- | --- | --- | --- | --- |
|  | *F* | *P* | *F* | *P* | *F* | *P* | *F* | *P* | *F* | *P* | *F* | *P* | *F* | *P* |
| SA | 40.148 | 0.008 | 7.087 | 0.016 | 85.677 | < 0.001 | 0.357 | 0.558 | 0.007 | 0.933 | 1.292 | 0.271 | 0.737 | 0.403 |
| Expression of *PR* | 191.672 | < 0.001 | 1.999 | 0.173 | 129.127 | < 0.001 | 0.460 | 0.505 | 7.137 | 0.015 | 1.698 | 0.207 | 3.182 | 0.09 |
| JA | 4.241 | 0.131 | 0.328 | 0.575 | 42.909 | < 0.001 | 0.433 | 0.52 | 0.790 | 0.387 | 0.007 | 0.971 | 0.026 | 0.875 |
| Expression of *PI* | 0.046 | 0.844 | 0.163 | 0.691 | 69.695 | < 0.001 | 0.570 | 0.461 | 0.977 | 0.337 | 0.106 | 0.749 | 0.209 | 0.654 |
| ABA | 16.775 | 0.029 | 304.106 | < 0.001 | 14.314 | 0.002 | 38.940 | < 0.001 | 0.009 | 0.926 | 28.907 | < 0.001 | 0.002 | 0.961 |
| Expression of *NCED1* | 21.406 | 0.019 | 125.676 | < 0.001 | 28.420 | < 0.001 | 9.115 | 0.008 | 0.040 | 0.845 | 14.634 | 0.001 | 0.378 | 0.547 |
| Expression of *SnRK2* | 25.797 | 0.014 | 88.460 | < 0.001 | 34.998 | < 0.001 | 15.756 | 0.001 | 30.808 | < 0.001 | 1.311 | 0.268 | 0.057 | 0.814 |

Table S.4 The callose content, enzyme activity, and fold-change in the expression of related genes involved in the callose synthesis and degradation for two tomato genotypes grown under ambient O_3_ and elevated O_3_ with *B. tabaci* infestation.

| Variable | O_3_ | | Plant genotype | | *B. tabaci* infestation | | O_3_ 🞨 Plant genotype | | O_3_ 🞨  *B. tabaci* infestation | | *B. tabaci* infestation 🞨 Plant genotype | | O_3_ 🞨 Plant genotype 🞨*B. tabaci* infestation | |
| --- | --- | --- | --- | --- | --- | --- | --- | --- | --- | --- | --- | --- | --- | --- |
|  | *F* | *P* | *F* | *P* | *F* | *P* | *F* | *P* | *F* | *P* | *F* | *P* | *F* | *P* |
| Callose content | 29.387 | 0.012 | 343.007 | < 0.001 | 94.220 | < 0.001 | 56.058 | < 0.001 | 4.972 | 0.040 | 69.141 | < 0.001 | 12.356 | 0.003 |
| Callose synthase activity | 38.191 | 0.008 | 236.929 | < 0.001 | 73.672 | < 0.001 | 43.794 | < 0.001 | 2.093 | 0.167 | 67.292 | < 0.001 | 2.054 | 0.171 |
| Expression of *Cals 11* | 95.118 | 0.002 | 373.764 | < 0.001 | 53.916 | < 0.001 | 40.783 | < 0.001 | 3.653 | 0.075 | 79.780 | < 0.001 | 4.637 | 0.048 |
| Expression of *Cals 12* | 65.442 | 0.004 | 332.411 | < 0.001 | 69.589 | < 0.001 | 56.971 | < 0.001 | 0.142 | 0.712 | 57.326 | < 0.001 | 2.920 | 0.107 |
| Beta-1,3 glucanase activity | 6.072 | 0.089 | 62.184 | < 0.001 | 62.209 | < 0.001 | 3.652 | 0.078 | 0.229 | 0.640 | 0.614 | 0.447 | 1.992 | 0.182 |
| Expression of *beta-1,3 glucanase* | 102.011 | 0.002 | 268.895 | < 0.001 | 130.046 | < 0.001 | 2.912 | 0.105 | 0.987 | 0.334 | 0.006 | 0.937 | 0.008 | 0.930 |

Table S.5 The callose content, enzyme activity, and fold-change in the expression of related genes involved in the callose synthesis and degradation for wild-type AC plants painting with ABA, 2-DGG, and H_2_O grown under ambient O_3_ and elevated O_3_ with *B. tabaci* infestation.

| Variable | O_3_ | | ABA treatment | | Callose inhibitor treatment | | *B. tabaci* infestation | | O_3_ 🞨  ABA treatment | | O_3_ 🞨  Callose inhibitor treatment | | O_3_ 🞨  *B. tabaci* infestation | |
| --- | --- | --- | --- | --- | --- | --- | --- | --- | --- | --- | --- | --- | --- | --- |
|  | *F* | *P* | *F* | *P* | *F* | *P* | *F* | *P* | *F* | *P* | *F* | *P* | *F* | *P* |
| Callose content | 335.474 | < 0.001 | 106.928 | < 0.001 | 422.073 | < 0.001 | 21.271 | < 0.001 | 0.952 | 0.336 | 25.080 | < 0.001 | 0.337 | 0.566 |
| Callose synthase activity | 61.402 | 0.004 | 39.210 | < 0.001 | 544.524 | < 0.001 | 53.893 | < 0.001 | 2.226 | 0.145 | 77.609 | < 0.001 | 0.001 | 0.978 |
| Expression of *Cals 11* | 82.918 | 0.002 | 62.509 | < 0.001 | 123.113 | < 0.001 | 707.920 | < 0.001 | 4.617 | 0.038 | 77.282 | < 0.001 | 4.213 | 0.047 |
| Expression of *Cals 12* | 51.571 | 0.006 | 70.751 | < 0.001 | 685.320 | < 0.001 | 126.036 | < 0.001 | 1.195 | 0.280 | 66.951 | < 0.001 | 0.886 | 0.352 |
| Beta-1,3 glucanase activity | 153.511 | 0.001 | 104.384 | < 0.001 | 0.789 | 0.381 | 138.972 | < 0.001 | 12.810 | 0.001 | 0.450 | 0.507 | 22.363 | < 0.001 |
| Expression of *beta-1,3 glucanase* | 92.826 | 0.002 | 103.150 | < 0.001 | 0.515 | 0.477 | 221.747 | < 0.001 | 0.610 | 0.439 | 0.181 | 0.673 | 15.829 | < 0.001 |

Table S.6 Population abundance and feeding behavior of *B. tabaci* when fed on wild-type AC plants with ABA, 2-DGG, and H_2_O under ambient O_3_ and elevated O_3_.

| Variable | O_3_ | | ABA treatment | | Callose inhibitor treatment | | O_3_ 🞨  ABA treatment | | O_3_ 🞨  Callose inhibitor treatment | | ABA treatment 🞨  Callose inhibitor treatment | | O_3_ 🞨 ABA treatment 🞨  Callose inhibitor treatment | |
| --- | --- | --- | --- | --- | --- | --- | --- | --- | --- | --- | --- | --- | --- | --- |
|  | *F* | *P* | *F* | *P* | *F* | *P* | *F* | *P* | *F* | *P* | *F* | *P* | *F* | *P* |
| The number of *B.tabaci* | 12.254 | 0.014 | 0.143 | 0.706 | 287.219 | < 0.001 | 1.776 | 0.185 | 0.580 | 0.448 | 11.832 | 0.001 | 0.954 | 0.331 |
| The total duration of NP | 0.190 | 0.664 | 0.034 | 0.854 | 2.125 | 0.148 | 0.595 | 0.442 | 0.122 | 0.728 | 0.084 | 0.773 | 1.483 | 0.226 |
| The total duration of C | 0.005 | 0.942 | 0.573 | 0.451 | 1.989 | 0.161 | 0.163 | 0.687 | 0.142 | 0.707 | 2.660 | 0.106 | 0.494 | 0.483 |
| The total duration of E1 | 15.348 | < 0.001 | 0.003 | 0.953 | 97.316 | 0.000 | 1.049 | 0.308 | 0.837 | 0.362 | 11.188 | 0.001 | .076 | 0.784 |
| The total duration of E2 | 7.283 | 0.008 | 8.723 | 0.004 | 186.899 | < 0.001 | 0.398 | 0.530 | 0.398 | 0.530 | 27.073 | < 0.001 | 0.924 | 0.339 |
| The total duration time to first E2 | 8.063 | 0.005 | 4.797 | 0.031 | 141.233 | < 0.001 | 0.092 | 0.762 | 6.519 | 0.012 | 11.596 | 0.001 | 0.030 | 0.862 |
| The total duration of G | 0.829 | 0.365 | 0.025 | 0.876 | 1.344 | 0.249 | 0.080 | 0.777 | 0.954 | 0.331 | 1.391 | 0.241 | 0.012 | 0.912 |
| The total duration of F | 0.083 | 0.774 | 1.701 | 0.195 | 0.793 | 0.375 | 0.010 | 0.921 | 0.843 | 0.361 | 0.006 | 0.941 | 0.710 | 0.401 |

Table S.7 Primer sequences used for real-time quantitative PCR.

| Gene | GenBank accession no. | Primer sequence（5’-3’） |
| --- | --- | --- |
| *NCED1*  *(9-cis-epoxycarotenoid dioxygenase)* | Solyc07g056570 | *F*:CTTATTTGGCTATCGCTGAACC  *R*:CCTCCAACTTCAAACTCATTGC |
| *SnRK2*  *(Sucrose non-fermenting 1-related protein kinase 2)* | SGN-U576095 | *F*: CAATGGCATACCACAATCCT  *R*: AGGGCTTCGCTTTGTAGTTG |
| *Cals11*  *callose synthase gene 11* | XM_ 010318449 | *F*: GAAGGACGAGAGAGAGATATGG  *R*: CTGAAGCAGAATCAAGGAACG |
| *Cals12*  *callose synthase gene 12* | XM_010325437 | *F*:TGAGGAGGCACTGAAAATGAGGAAC  *R*: CGGATTTTCAGGGGGTTGGCT |
| *beta-1, 3-glucanase* | LOC543986 | *F*: GCGGTGTTCAGCCTGGATG  *R*: AGCATGAGCAAGAAGTATGTTGTG |
| *TIP41* | SGN-U321250 | *F*: AGGCCTTGTCTTCGAAAGGA  *R*: TCCTTTAGGACACTCCAACATGG |
| *Actin* | AB199316 | *F*: TGGTCGGAATGGGACAGAAG  *R*: CTCAGTCAGGAGAACAGGGT |

Fig. S.1 A summary of the whole experimental design in the current study.


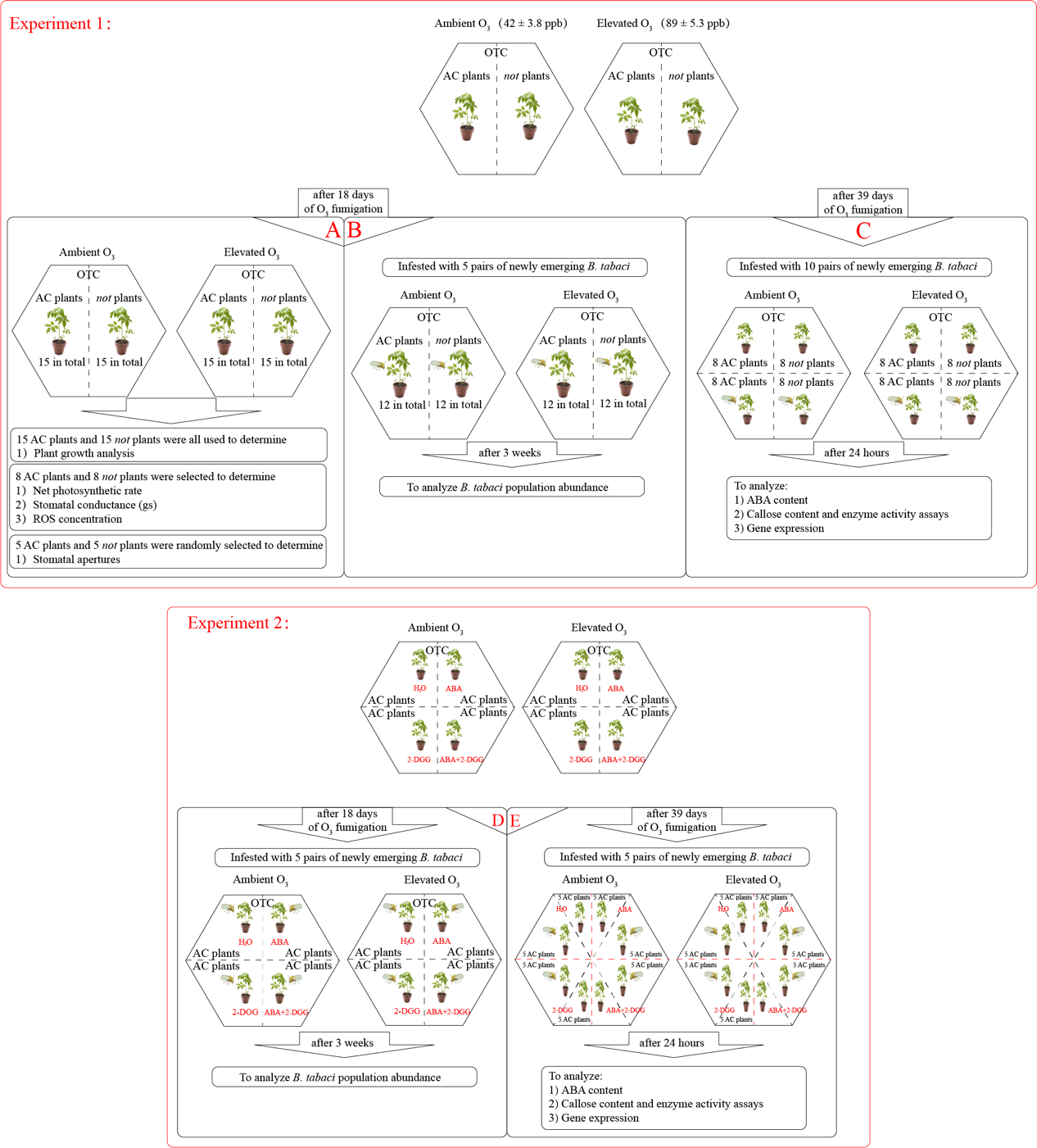


Fig. S.2 ABA content and relative expression of related genes involved in the ABA signaling pathway for wild-type AC plants treating with H_2_O, ABA, 2-DGG, and ABA plus 2-DDG grown under ambient O_3_ and elevated O_3_ with and without *B. tabaci* infestation. Each value represents the average (± SE) of four OTCs (eight plants for each genotype per OTC). (A) ABA content, (B) The relative expression of *NCED1*, (C) The relative expression of *SnRK2*. Different lowercase letters indicate significant differences among the combinations of *B. tabaci* treatment and O_3_ concentrations within the same genotype. Different uppercase letters indicate significant differences between genotypes within the same O_3_ treatment and *B. tabaci* treatment as determined by Tukey’s multiple range test at *P* < 0.05.


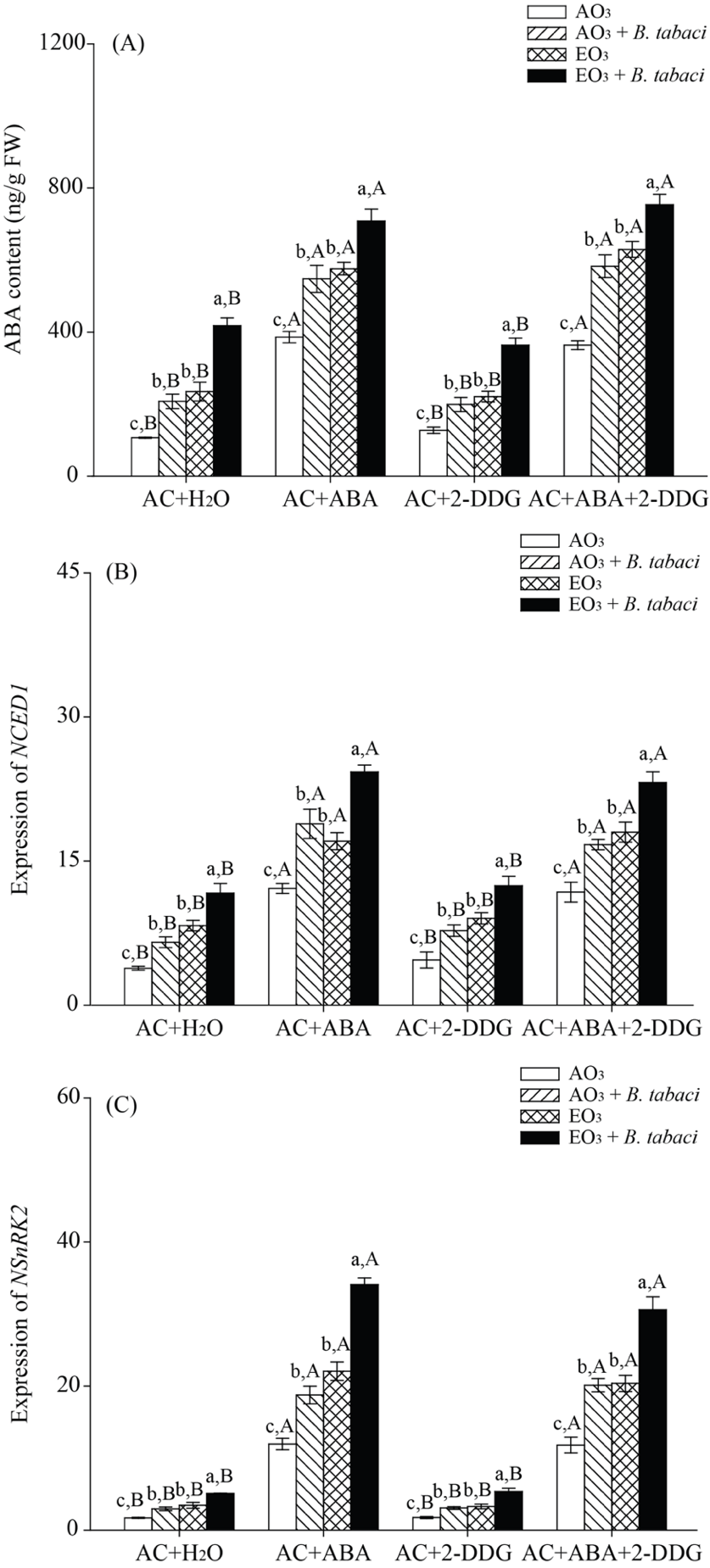

Supplement: Supplementary file 1 [file DataSheet_1.docx]
